# Perspectives on Data Sharing in Persons With Spinal Cord Injury

**DOI:** 10.1089/neur.2023.0035

**Published:** 2023-11-09

**Authors:** Freda M. Warner, Bobo Tong, Jessie McDougall, Kathleen A. Martin Ginis, Alexander G. Rabchevsky, Jacquelyn J. Cragg, John L.K. Kramer

**Affiliations:** ^1^ International Collaboration on Repair Discoveries (ICORD), Blusson Spinal Cord Centre, University of British Columbia, Vancouver, British Columbia, Canada.; ^2^ Department of Rehabilitation Sciences, Faculty of Medicine, University of British Columbia, Vancouver, British Columbia, Canada.; ^3^ Division of Physical Medicine and Rehabilitation, Department of Medicine, University of British Columbia, Kelowna, British Columbia, Canada.; ^4^ School of Health and Exercise Sciences, Faculty of Health and Social Development, University of British Columbia, Kelowna, British Columbia, Canada.; ^5^ Department of Physiology and Spinal Cord and Brain Injury Research Center (SCoBIRC), University of Kentucky, Lexington, Kentucky, USA.; ^6^ Faculty of Pharmaceutical Sciences, University of British Columbia, Vancouver, British Columbia, Canada.; ^7^ Department of Anesthesiology, Pharmacology, and Therapeutics, Faculty of Medicine, University of British Columbia, Vancouver, British Columbia, Canada.

**Keywords:** data privacy, data sharing, informed consent, open science, spinal cord injury

## Abstract

Open data sharing of clinical research aims to improve transparency and support novel scientific discoveries. There are also risks, including participant identification and the potential for stigmatization. The perspectives of persons participating in research are needed to inform open data-sharing policies. The aim of the current study was to determine perspectives on data sharing in persons with spinal cord injury (SCI), including risks and benefits, and types of data people are most willing to share. A secondary aim was to examine predictors of willingness to share data. Persons with SCIs in the United States and Canada completed a survey developed and disseminated through various channels, including our community partner, the North American Spinal Cord Injury Consortium. The study collected data from 232 participants, with 52.2% from Canada and 42.2% from the United States, and the majority completed the survey in English. Most participants had previously participated in research and had been living with an SCI for ≥5 years. Overall, most participants reported that the potential benefits of data sharing outweighed the negatives, with persons with SCI seen as the most trustworthy partners for data sharing. The highest levels of concern were that information could be stolen and companies might use the information for marketing purposes. Persons with SCI were generally supportive of data sharing for research purposes. Clinical trials should consider including a statement on open data sharing in informed consents to better acknowledge the contribution of research participants in future studies.

## Introduction

Open data sharing is now commonplace across clinical research.^1–3^ This reflects the desire to increase transparency and rigor in biomedical research and support novel inquiries through secondary analyses.^2,3^ Risks of data sharing include participant reidentification^4,5^ and subsequent stigmatization, as well as the proliferation of so-called “junk science”^3,6^ and “data parasites”.^7^ The extent to which the benefits of sharing clinical data outweigh the negatives has primarily been discussed in academic forums, with little input directly from clinical trial participants.^8^

Spinal cord injury (SCI) is a complex neurological condition caused by damage to the spinal cord, characterized by varying levels of lifelong muscle paralysis, sensory impairments, autonomic deficits, and secondary complications.^9,10^ Reflecting this complexity, persons with SCI participate in research that collects highly specific and sensitive data, such as that pertaining to bowel, bladder, sexual, and mental health.^10^ Though SCI is a relatively rare condition, burden to the person and societal costs are high.^11^ This highlights the importance of increased efforts toward data sharing and utilization in SCI research to fully understand the complexities of the condition. However, the willingness and preferences of persons with SCI to share their data have not yet been explored.

In support of developing a framework for data sharing, we sought to examine perspectives from persons with SCIs. Our specific aim was to determine the overall perspective on data sharing, including its risks and benefits, and types of data that people were most willing to share. A secondary aim was to examine predictors of willingness to share data.

## Methods

### Participants

Perspectives were solicited from persons with traumatic and non-traumatic SCIs of any age and identified gender, residing in the United States or Canada. The study was approved by the University of British Columbia Clinical Research Ethics Board (H18-03693).

### Survey development

The preliminary survey was guided, in part, by an original questionnaire delivered to clinical trial participants in a study published by the *New England Journal of Medicine*.^12^ Questions specific to our research were developed by experts in the field and primarily focused on SCI-specific issues and health concerns. This survey was first reviewed by study team members and then beta tested by key community partners and persons with SCIs at the North American Spinal Cord Injury Consortium (NASCIC), followed by a focus group discussion. This feedback was reviewed and integrated, and a final version of the survey was prepared for large-scale dissemination in English, French, and Spanish (see Supplementary Material).

### Survey dissemination

Initially, information regarding the survey was posted in two NASCIC monthly newsletters and NASCIC's social media to recruit interested participants. Identical information was also posted on the website for the International Collaboration on Repair Discoveries (ICORD),^13^ as well as on social media (Twitter and Facebook) by official ICORD and Praxis (formerly the Rick Hansen Institute) accounts. Spinal Cord Injury BC also advertised the survey through its provincial and national communication channels.

Additionally, persons who had previously participated in Praxis' Spinal Cord Injury Community Survey and consented to future communication were contacted by e-mail.^14^ This e-mail was also forwarded to persons in the research list serve of the Miami Project.^15^ All participants were compensated with gift cards for completing the survey.

Interested participants were required to contact a researcher (F.M.W.) by phone or by e-mail confirming that they fulfilled the participation criteria. Because of the open nature of the initial recruitment tactic, participants who opted to communicate by e-mail were required to answer additional questions (e.g., injury-specific queries) aimed at confirming their status as a valid participant. Eligible participants were then provided with a secure and unique web link by e-mail to complete the survey using Qualtrics software (Qualtrics LLC, Provo, UT).^16^ In processing the data, we applied Qualtrics' internal survey protection tools to identify likely bots and duplicate responses (e.g., reCAPTCHA and duplicate scores), which were removed before analysis.^17^

### Statistical analysis

All survey responses were recorded in Qualtrics and output onto a spreadsheet. No responses were linked to participant names or e-mails, and all were deidentified by Qualtrics and assigned a random identification number. Descriptive statistics were reported for the results of the survey, summarizing demographics and attitudes toward data sharing. Multi-variable logistic regression models were used to determine associations between potential demographic predictors and data-sharing attitudes. Analyses were performed using RStudio.^18^ Further information on model construction can be found in the Supplementary Material.

## Results

### Sample characteristics

Data collection was conducted from July 2020 to August 2021. Unique survey e-mail links were sent to a total of 335 participants: 108 by initial open recruitment methods, 151 from the Praxis list serve, and 76 from the Miami Project list serve. A total of 287 online responses were returned, of which 229 were deemed valid. In addition, three phone responses were included, totaling 232 valid participants. Of these valid responses, 52.2% were living in Canada and 42.2% were in the United States (Table 1). The majority of surveys were completed in English (96.6%). Most participants indicated that they had previously participated in research (61.6%; Table 1) and been living with an SCI for ≥5 years (68.1%; Table 2).

**Table 1.** Sample Characteristics

Characteristic	*N* (%^*^)
Total	232 (100)
Sex	
Male	133 (57.3)
Female	85 (36.6)
Did not/prefer not to respond	14 (6.0)
Age, years	
≤49	106 (45.7)
>49	102 (44.0)
Did not/prefer not to respond	24 (10.3)
Country	
Canada	121 (52.2)
United States	98 (42.2)
Did not/prefer not to respond	13 (5.6)
Race (can select more than 1)	
White	173 (74.6)
Black or African American	7 (3.0)
Other	30 (12.9)
Did not/prefer not to respond	9 (3.9)
Education	
Less than high school	5 (2.2)
High school diploma	39 (16.8)
Diploma or certificate from trade, technical, or vocational school, or college or CEGEP	66 (28.4)
Bachelor or undergraduate degree, or teacher's college	68 (29.3)
Graduate degree	39 (16.8)
Did not/prefer not to respond	15 (6.5)
Health status	
Poor	12 (5.2)
Fair	38 (16.4)
Good	95 (40.9)
Very good	59 (25.4)
Excellent	15 (6.5)
Did not/prefer not to respond	13 (5.6)
Previous participation in research	
Yes	143 (61.6)
No	84 (36.2)
Did not/prefer not to respond	5 (2.2)

^*^ Percentage may not add up to 100% due to rounding; CEGEP, Collège d'enseignement général et professionnel (general and professional teaching college in English).

**Table 2.** SCI Characteristics

Characteristic	*N* (%^*^)
Years living with SCI	
≤1	1 (0.4)
2	4 (1.7)
3	3 (1.3)
4	4 (1.7)
≥5	158 (68.1)
Did not/prefer not to respond	62 (26.7)
Level of injury	
Lower back (lumbar spine)	32 (13.8)
Mid back (lower thoracic spine)	65 (28.0)
Upper back (upper thoracic spine)	30 (12.9)
Neck (cervical spine)	90 (38.8)
Did not/prefer not to respond	15 (6.5)
Classification of injury	
Paraplegia	122 (52.6)
Quadriplegia	92 (39.7)
Did not/prefer not to respond	18 (7.8)
SCI etiology	
Non-traumatic	42 (17.9)
Traumatic	177 (76.3)
Did not/prefer not to respond	13 (5.6)
Type of mobility aid (can select more than one)	
I walk without the help of a special aide, tool, or person	16 (6.9)
I walk with the help of a special aide, tool, or person	63 (27.2)
I use a manual wheelchair	120 (51.7)
I use a power wheelchair or scooter	60 (25.9)
Other	8 (3.4)
Did not/prefer not to respond	13 (5.6)

^*^ Percentage may not add up to 100% due to rounding.

SCI, spinal cord injury.

**Table 3.** Willingness of Participants to Share Their Data According to Type of Use and Recipient

	Very unlikely (%)	Somewhat unlikely (%)	Neither likely not unlikely (%)	Somewhat likely (%)	Very likely (%)	Did not respond (%)
Type of use (abbreviated)						
Scientific accuracy	4 (1.7)	5 (2.2)	22 (9.5)	45 (19)	144 (62)	12 (5.2)
Inform persons with SCIs about health	5 (2.2)	14 (6.0)	8 (3.4)	31 (13)	162 (70)	12 (5.2)
Research my/family's health problems	1 (0.0)	8 (3.4)	22 (9.5)	40 (17)	149 (64)	12 (5.2)
Faster scientific answers	3 (1.3)	10 (4.3)	21 (9.1)	40 (17)	146 (63)	12 (5.2)
Research to help others	3 (1.3)	6 (2.6)	17 (7.3)	39 (17)	154 (66)	13 (5.6)
Lawyers proving medical lawsuits	17 (7.3)	18 (7.8)	74 (31.7)	49 (21)	62 (27)	12 (5.2)
Learn about rare diseases	2 (0.9)	4 (1.7)	32 (14)	49 (21)	133 (57)	12 (5.2)
Recipient (abbreviated)						
Scientists in universities	3 (1.3)	3 (1.3)	22 (9.5)	42 (18)	148 (63.4)	14 (6.0)
Persons with SCIs	8 (3.4)	9 (3.9)	14 (6.0)	30 (13)	158 (68.4)	13 (5.6)
Companies developing medical products	3 (1.3)	10 (4.3)	20 (8.6)	73 (31)	113 (48.4)	13 (5.6)
Doctors	0 (0.0)	5 (2.2)	26 (11)	41 (18)	147 (63.4)	13 (5.6)
Insurance companies	16 (6.9)	17 (7.3)	62 (27)	56 (24)	67 (28.4)	14 (6.0)
Government agencies	7 (3.0)	19 (8.2)	50 (22)	60 (26)	81 (34.4)	15 (6.5)

### Perceived risks of data sharing

**FIG. 1.**
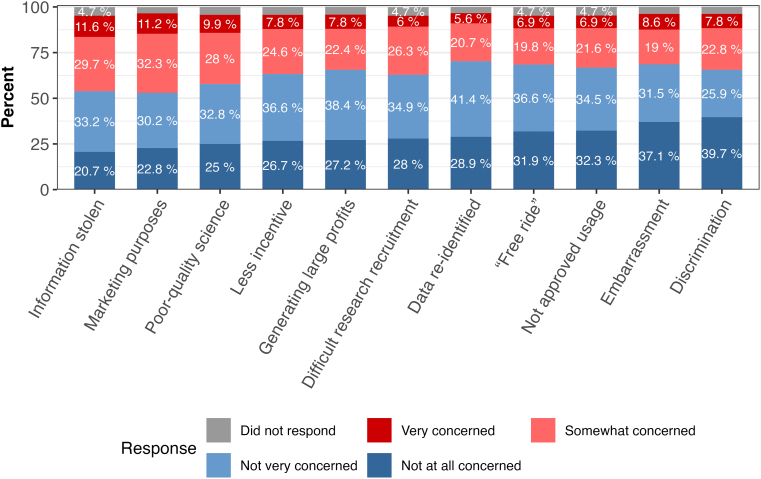
Level of concern regarding potential consequences of data sharing. The x-axis represents different concerns for data sharing, including: information stolen: the information might be stolen; marketing purposes: companies might use the information for marketing purposes instead of scientific purposes; poor-quality science: people might use the data to do poor-quality science; less incentive: scientists and companies might have less incentive to invest time and money in doing research studies; generating large profits: some person or company could make a lot of money developing products using a person's information; difficult research recruitment: it could be harder to get people to agree to be in research studies if they know their data will be shared; data reidentified: someone who is good with computers could identify the data; “free ride”: scientists or companies could unfairly “free ride” on the work of others; not approved usage: the information might be used in scientific projects that the participants would not approve of; embarrassment: people could be embarrassed if the information was linked back to them; and discrimination: people could be discriminated against if the information was linked back to them.

The majority of participants (>50%) were “not at all” or “not very” concerned regarding any of the potential negatives arising from data sharing (Fig. 1; Supplementary Table S1). The issues recording the highest levels of concern were that information could be stolen (41.3%) or that companies might use the information for marketing purposes (43.5%; Supplementary Table S1). When asked to select the most important potential negative of data sharing, poor-quality science (13.9%), stolen information (12.6%), and use of information for marketing purposes (12.6%) were the top three responses (Supplementary Table S2).

### Perceived benefits of data sharing

**FIG. 2.**
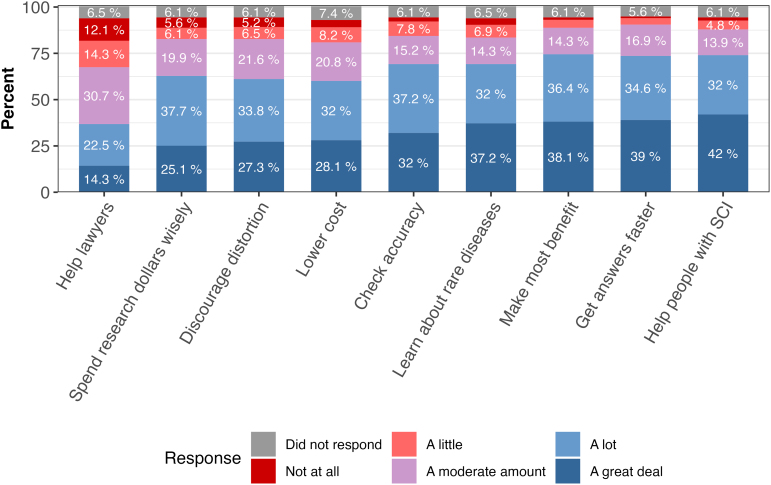
How much sharing anonymous, individual research data could lead to potential benefits. The x-axis represents different potential benefits of data sharing, including: help lawyers: can help lawyers prove their case in lawsuits claiming that medical products are unsafe; spend research dollars wisely: can help ensure that research dollars are spent as wisely as possible; discourage distortion: can discourage scientists and companies from hiding or distorting their research study results (by making it possible for others to check their analyses); lower cost: can lower the cost of developing new medical products; check accuracy: can help scientists check the accuracy of research results announced by other scientists or companies (by redoing the analyses); learn about rare diseases: can support learning about diseases that only a rare number of people have (by combining data from many research studies); make the most benefit: can make sure a person's participation in research studies leads to the most scientific benefit; get answers faster: can help get answers to scientific questions faster using information that others have already gathered; and help persons with SCI: can help persons living with SCIs learn more about health problems that affect them. SCI, spinal cord injury.

When asked who would benefit “a lot” or “a great deal” from data sharing, participants indicated that scientists and doctors would benefit the most (78.0% and 76.8%) and health insurance companies the least (52.2%; Supplementary Table S3). In line with these findings, most participants believed that data sharing would lead to a list of potential benefits “a lot” or “a great deal.” The exception to this was the potential that data sharing would help lawyers with lawsuits (8.4% indicated “a lot” or “a great deal”; Fig. 2; Supplementary Table S4). Participants identified the most important benefits of data sharing to be helping to get faster answers to scientific questions using information that others have already gathered (25.0%) and helping patients learn more about health problems that affect them (17.7%; Supplementary Table S5).

### Overall support for data sharing

Overall, the majority (79.3%) of participants believed that the potential benefits of data sharing outweighed the negatives. Few (6.9%) perceived that the negative aspects outweighed the benefits or that the risks and benefits were equal (8.2%; Supplementary Table S6).

With regard to trusted data-sharing partners, participants had “a great deal” or “a lot” of trust in persons living with SCIs (72.8%), followed by doctors (67.2%) and scientists (66.0%). Conversely, the lowest levels of trust were recorded for government agencies (26.3%) and health insurance companies (21.5%; Supplementary Table S6). Participants' willingness to share their data with partners mirrored their level of trust, given that they were most willing to share with scientists, persons with SCIs, companies developing medical products, and doctors (80–82%) and least likely with government and insurance agencies (53–81%; Supplementary Table S7).

When participants were queried about the kind of permission required for sharing data, they were fairly evenly divided between not needing to provide permission beyond the initial consent (36.2%) and formally asking permission, separate from their decision to be involved in the original study (34%). Almost one quarter of participants also indicated the desire not only to be formally asked permission to share their research data, but also to be asked every time the data would be accessed by others. Less than 3% of participants indicated that they did not want their data shared at all (Supplementary Table S8). Of those preferring permission to be asked, the majority indicated that it was because “it's part of showing respect to participants” (44.0%), whereas others believed it was because of the risk it placed to participants (30.2%; Supplementary Table S9). However, if permission could not be obtained from original participants, 69.0% indicated that it would be acceptable to share anonymous data (Supplementary Table S10). More than half of participants also indicated that they did not require financial compensation if their individual data were shared (Supplementary Table S11).

### Differences in data sharing related to the type of data

**FIG. 3.**
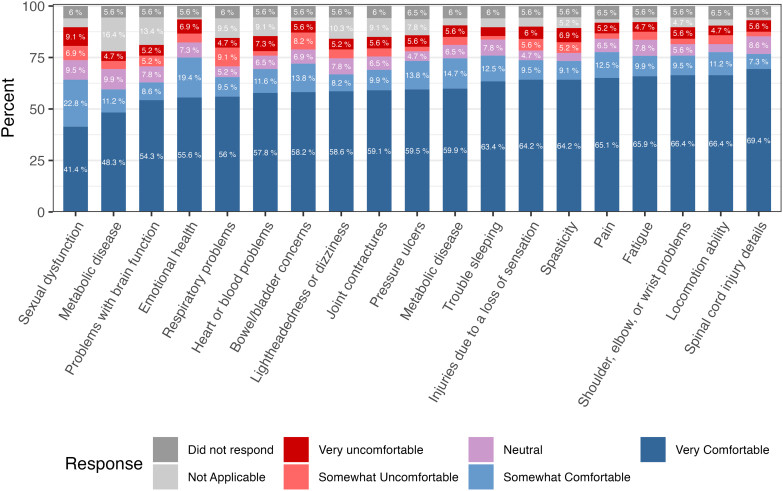
Level of comfort regarding the sharing of specific types of data.

The majority of participants were “very” or “somewhat comfortable” sharing details about all the secondary health issues outlined in the survey. The highest levels of “very” or “somewhat uncomfortable” responses were reported for details regarding sexual dysfunction (16.0%), bladder/bowel concerns (13.8%), and respiratory problems (13.8%; Fig. 3; Supplementary Table S12).

### Predictors of data-sharing attitudes

The odds of participants reporting that the negative aspects of data sharing outweighed the benefits were decreased among those who had college and/or graduate degrees (odds ratio [OR], 0.14; 95% confidence interval [CI], 0.03–0.50), and previously participated in research (OR, 0.09; 95% CI, 0.01–0.43), and was significantly higher among those who were concerned about the risk of misappropriation (OR, 10.3; 95% CI, 1.64–206; Supplementary Table S13).

## Discussion

Among persons with SCI, nearly 80% indicated support for data sharing, such that the benefits outweighed the negatives. Poor-quality science, stolen information, and companies co-opting information for marketing purposes were ranked as top concerns, whereas answering scientific questions faster and helping patients were the top perceived benefits. Persons with SCI were most willing to trust and share their data with other persons with SCI, as well as doctors and scientists. The type of data impacted participants' data-sharing perspectives, with traditionally sensitive topics associated with the highest levels of discomfort. A substantial proportion of participants preferred that researchers request permission to share data; however, the majority were supportive of anonymous data sharing when permission could not be obtained and did not request financial compensation. General perspectives on data sharing were influenced by education and past participation in research, as well as concerns for misappropriation.

The overall level of support for data sharing among persons with SCI was similar to that reported in the original survey, which broadly sampled clinical trial participants.^12^ These findings were also in line with regard to the perceived benefits, recipients, and types of use. The results of the multi-variable regression of data sharing only overlapped on the important role of graduate education and is supported elsewhere.^12,19,20^ The lack of emphasis on the need to be compensated for data sharing is consistent with the notion that many persons participate in research for altruistic purposes.^21,22^

Differences emerged, however, with regard to potential negatives. Foremost, the proportion of those who were “somewhat” or “very” concerned about potential negatives of data sharing was higher than those surveyed by Mello and colleagues for 9 of 11 issues.^12^ Closer inspection revealed the impact of SCI on data-sharing perspectives. For example, the greatest discrepancies occurred for the concerns about “embarrassing” data being linked back to them (28% vs. 14%) and the conduct of poor-quality science (38% vs. 24%). These differences may be attributable to the health of respondents, given that Mello and colleagues surveyed mostly “healthy” participants. Compared to “healthy controls,” participants living with a disease are easier to identify (e.g., owing to the phenotype of their disease) and may be more likely to have provided sensitive and stigmatizing data. In addition to the risk of identification, the potential for poor-quality science to emerge using shared data stands to affect these participants directly (e.g., through changes in their medical management), which is not the case for “healthy controls.”

Our results also demonstrated that concerns for clinical data sharing are proportionate to the sensitivity of the data proposed to share. This was important to determine because persons with SCI engage in various types of research and, in many cases, will be asked to divulge sensitive data. Nevertheless, most respondents were very or somewhat comfortable sharing data regardless of type, thereby suggesting little need to impose specific limitations.

Waivers of consent have served as the ethical backbone for sharing historical clinical trial data. Such a waiver is considered ethically justified so long as data can be deidentified or completely anonymized, poses minimal risk to participants, and if, for various reasons, the research could not proceed without a waiver.^23^ The majority of participants accepted the concept of a waiver of consent; however, most preferred a model that sought permission beforehand. Interestingly, the value of seeking permission was related to the importance of demonstrating respect toward research participants, rather than risk of identification. To respond to this preference, future clinical trials should implement consent processes that allow for sharing and secondary uses of data.

A strength of our study is that the cohort represents persons with SCIs living in both Canada and the United States. Further, we used multiple methods for disseminating the survey (i.e., mail, e-mail, or phone), in multiple languages, through well-established spinal cord community channels. A limitation to our study is the demographic profile of our cohort. The majority of participants identified as white and were almost all English speakers, and the majority had chronic traumatic SCI, which could affect the generalizability of our findings. Moreover, these findings may not be applicable outside of the United States/Canada, and may not be applicable to developing countries, where “helicopter research”^24^ may have enhanced concerns regarding data sharing.

Our survey provides a snapshot into the perspectives of persons with SCIs on the sharing of health data. Overall, participant views of data sharing are positive and invested in how their data will be used, if openly shared. This information should be incorporated as we move forward in the creation of new data-sharing platforms and expansion of existing open data platforms, such as the Open Data Commons for Spinal Cord Injury,^25^ and brings us one step closer to the design of a patient-oriented, open access, clinical trial repository of research participant data. These findings are also relevant to policies and legislation that have posed challenges to secondary uses of data, such as the European Union's General Data Protection Regulation.^26^

## Supplementary Material

Supplemental data

Supplemental data

Supplemental data

Supplemental data

Supplemental data

Supplemental data

Supplemental data

Supplemental data

Supplemental data

Supplemental data

Supplemental data

Supplemental data

Supplemental data

Supplemental data

## Abbreviations Used

CI: confidence interval
ICORD: International Collaboration on Repair Discoveries
NASCIC: North American Spinal Cord Injury Consortium
OR: odds ratio
SCI: spinal cord injury
