# Supplementary material for: Perspectives on Data Sharing in Persons With Spinal Cord Injury
Source: Neurotrauma Rep. 2023 Nov 9;4(1):781–9. doi: 10.1089/neur.2023.0035 (PMC10659015; doi:10.1089/neur.2023.0035)
Supplement: Supplemental data [file Suppl_Material.zip › English SCI Data Share Survey.docx]

Welcome to the

Spinal Cord Injury Research Participants' Views on Data Sharing Survey

Conducted by:

The Kramer Lab, ICORD

University of British Columbia, Vancouver, B.C.

In partnership with:

North American Spinal Cord Injury Consortium (NASCIC)

Primary Investigator: Dr. John Kramer

International Collaboration on Repair Discoveries (ICORD)

818 West 10th Avenue

Vancouver, BC V5Z 1M9

John.kramer@ubc.ca


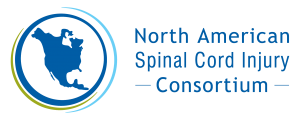

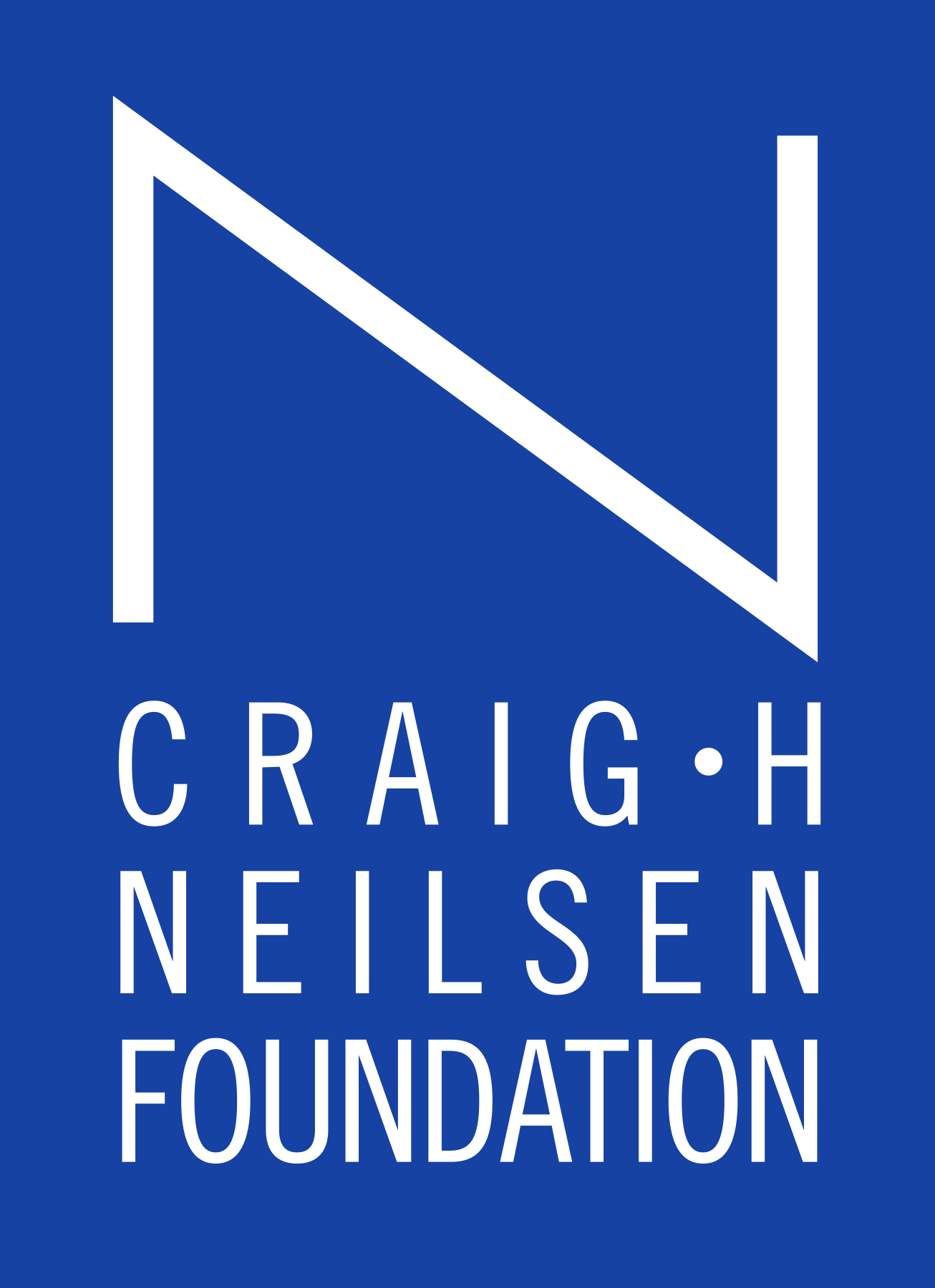


**Why are we doing this survey?**

You have been invited to participate in a research study investigating how individuals with spinal cord injury (SCI) feel about researchers using their data. Specifically, we are interested in learning more about your perceptions about ***data sharing***.

***Data sharing*** is when the data collected in one research study is **anonymously** shared with others. This means that a researcher allows others to see and use the individual data they collected in a research study, after deleting information that could identify people (like your name and exact birthdate). However, other personal information like your age range or medical information may be provided in full.

**We want to know how you feel about data sharing**. As it is your information that would be shared, we want to know your thoughts on this topic. Hopefully the results of this study will help to make decisions on if data should be shared, and how it should be done. Whether you have participated in a research study or will in the future, your opinion is important to us. But please be aware, **your participation in this survey, and your responses to the survey, will not affect what happens to your own research data.**

**What does taking the survey involve?**

If you decide to participate, you will be asked to fill out the following survey that should take approximately 30 minutes. It asks questions about your health and spinal cord injury status, and for your opinion on the potential benefits and risks of data sharing, your preferences regarding informed consent and privacy protection, and your level of trust in researchers and others. We are collecting information regarding your health and injury to find out about the characteristics of the people who took this survey.

You are free to skip any questions you do not want to answer, and you may stop the survey at any time. The data collected from this survey will be stored in an encrypted password-protected file on UBC’s secure servers. If you decide to provide us with your email, this information will be kept separate from your survey in an encrypted password-protected file on UBC’s secure servers. This means that once you submit your survey you will not be able to withdraw your data, as it will have no identifying information attached to it. If you share your email with us and want to find out the results of the study, we will provide you with a summary of the study’s findings.

**Risks and benefits**

A benefit of participating in this study is the opportunity to add your voice to the debate around data sharing, and potentially inform future data sharing decisions. We are also offering a $10 gift card for participation. The results of the surveys are completely anonymous.

**Your rights:**

If you have decided to participate in this project, please understand:

- Your participation is voluntary and you can withdraw your consent or stop participating at any time without penalty.
- Your ability to continue participating in research (if you are currently a participant) will not be affected by your participation in this survey study.
- The results of this study may be presented at scientific meetings or be published in scientific journals, and the survey data may be shared with other researchers. However, identifying information will not be disclosed.

**If you have questions:**

If you have any concerns or complaints about your rights as a research participant and/or your experiences while participating in this study, contact the Research Participant Complaint Line in the University of British Columbia Office of Research Ethics by e-mail at RSIL@ors.ubc.ca or by phone at 604-822-8598 (Toll Free: 1-877-822-8598).

| **Statement of Consent:** |
| --- |
| **By completing and returning the survey, you are giving your informed consent to participate in the study.** |

# **Survey on Sharing Data from Spinal Cord Injury Research Studies**

1. Who is completing this survey?
   1. I am completing this survey for myself
   2. I am completing this survey as a parent or guardian on behalf of a child
   3. I am completing this on behalf of another adult
   4. Other: _____

If you are completing this survey on behalf of another person, please answer these questions about them. If you are completing this survey for yourself, please answer them about yourself.

1. Do you have a spinal cord injury?
   1. Yes
   2. No

We would like to ask you about your **most recent participation** in a **research study**, which could have taken place either at a university, college, hospital, clinic, at your home, or in the community.

1. Have you participated in research prior to this survey?
   1. Yes
   2. No (skip to question 6)
2. Thinking about the most recent research study you participated in, what was the most important reason you decided to be in the study? (CHECK ONE)
   1. I thought there was a chance I might get a health benefit
   2. I wanted to help others
   3. I valued the chance to make some money
   4. Some other reason (please write):
3. Overall, how would you describe your experience as a research study participant?
   1. Very positive
   2. Somewhat positive
   3. Neither positive nor negative
   4. Somewhat negative
   5. Very negative

| We are also interested in your opinion on **research data sharing**. What do we mean by this?  ***Data sharing*** refers to the sharing of **anonymous** research study data with others who were not a part of the original research. This data has all personal identifiers removed (such as names or exact birthdates) before being shared. People that could access this data could include other researchers, companies developing medical products, patients, doctors, government officials, and others.  Data sharing refers to sharing anonymous information about each individual research participant, not just the results of the study. This means that all information collected from you in a study (for example, your age range, type of SCI, level of SCI, and your outcome of the study) would be shared. This could also include genetic information. |
| --- |

1. Some people have concerns about sharing anonymous, individual research data. Listed below are some of these concerns, how concerned are you about these issues? (CONTINUED ON NEXT PAGE)

|  | Not at all concerned | Not very concerned | Somewhat concerned | Very concerned |
| --- | --- | --- | --- | --- |
| 1. Someone who is good with computers could identify the data |  |  |  |  |
| 1. People could be discriminated against if the information was linked back to them |  |  |  |  |
| 1. People could be embarrassed if the information was linked back to them |  |  |  |  |
| 1. People might use the data to do poor-quality science |  |  |  |  |
| 1. The information might be used in scientific projects that the participants wouldn’t approve of |  |  |  |  |
| 1. Some person or company could make a lot of money developing products using people’s information |  |  |  |  |
| 1. It could be harder to get people to agree to be in research studies if they know their data will be shared |  |  |  |  |
| 1. The information might be stolen |  |  |  |  |
| 1. Companies might use the information for marketing purposes instead of scientific purposes |  |  |  |  |
| 1. Scientists or companies could unfairly “free ride” on the work of others |  |  |  |  |
| 1. Scientists and companies might have less incentive to invest time and money in doing research studies. |  |  |  |  |
| 1. Other concern (please write): |  |  |  |  |

1. From that list of potential concerns regarding sharing anonymous, individual research data, which is the ONE most important concern? Please circle it in Question 4 above.
2. Although there are concerns about sharing anonymous, individual research data, there are several potential benefits. How much do you think sharing anonymous, individual research data could lead to these potential benefits?

|  | Not at all | A little | A moderate amount | A lot | A great deal |
| --- | --- | --- | --- | --- | --- |
| 1. Can help get answers to scientific questions faster using information that others have already gathered. |  |  |  |  |  |
| 1. Can help ensure that research dollars are spent as wisely as possible. |  |  |  |  |  |
| 1. Can lower the cost of developing new medical products. |  |  |  |  |  |
| 1. Can help people living with spinal cord injuries learn more about health problems that affect them. |  |  |  |  |  |
| 1. Can help scientists check the accuracy of research results announced by other scientists or companies (by re-doing the analyses). |  |  |  |  |  |
| 1. Can support learning about diseases that only a small number of people have (by combining data from many research studies). |  |  |  |  |  |
| 1. Can discourage scientists and companies from hiding or distorting their research study results (by making it possible for others to check their analyses). |  |  |  |  |  |
| 1. Can help lawyers prove their case in lawsuits claiming that medical products are unsafe. |  |  |  |  |  |
| 1. Can make sure people’s participation in research studies leads to the most scientific benefit possible. |  |  |  |  |  |
| 1. Other benefit (please write): |  |  |  |  |  |

1. From that list of potential benefits of sharing anonymous, individual research data, which is the ONE most important benefit? Please circle it in Question 6 above.
2. Which of the following personal information would you feel comfortable being shared as anonymous, individual research data? (CONTINUED ON NEXT PAGE)

|  | Very comfortable | Somewhat comfortable | Neutral | Somewhat uncomfortable | Very uncomfortable | Not Applicable |
| --- | --- | --- | --- | --- | --- | --- |
| 1. Details about your spinal cord injury (e.g. how it occurred) |  |  |  |  |  |  |
| 1. Locomotion ability (e.g. walking) |  |  |  |  |  |  |
| 1. Emotional health (e.g. happiness, depression) |  |  |  |  |  |  |
| 1. Sexual dysfunction |  |  |  |  |  |  |
| 1. Spasticity (Spontaneous and uncontrolled, jerky muscle movements, such as uncontrolled muscle twitch   or spasm) |  |  |  |  |  |  |
| 1. Pain |  |  |  |  |  |  |
| 1. Bowel/bladder concerns (e.g. incontinence, kidney stones, urinary tract infections, constipation) |  |  |  |  |  |  |
| 1. Joint contractures (one or more joints that are frozen or have limited range of motion) |  |  |  |  |  |  |
| 1. Shoulder, elbow, or wrist problems (e.g. pain in these joints) |  |  |  |  |  |  |
| 1. Metabolic disease (e.g. diabetes) |  |  |  |  |  |  |
| 1. Weight problems |  |  |  |  |  |  |
| 1. Heart or blood problems (e.g. high blood pressure, autonomic dysreflexia, clots) |  |  |  |  |  |  |
| 1. Trouble sleeping |  |  |  |  |  |  |
| 1. Respiratory problems (e.g. pneumonia) |  |  |  |  |  |  |
| 1. Pressure ulcers |  |  |  |  |  |  |
| 1. Problems with brain function (e.g. talking, understanding words, memory, vision) |  |  |  |  |  |  |
| 1. Fatigue |  |  |  |  |  |  |
| 1. Injuries that are due to a loss of sensation in that body part |  |  |  |  |  |  |
| 1. Lightheadedness or dizziness |  |  |  |  |  |  |
| 1. Other concern (please write): |  |  |  |  |  |  |

1. How much do you think the following groups could benefit from sharing anonymous, individual research data?

|  | Not at all | A little | A moderate amount | A lot | A great deal |
| --- | --- | --- | --- | --- | --- |
| 1. Scientists in universities and other not-for-profit organizations |  |  |  |  |  |
| 1. People living with spinal cord injuries |  |  |  |  |  |
| 1. Companies developing medical products, such as prescription drugs |  |  |  |  |  |
| 1. Doctors taking care of patients |  |  |  |  |  |
| 1. Health insurance companies |  |  |  |  |  |
| 1. Government agencies |  |  |  |  |  |

1. How likely would you be to allow your anonymous, individual research data to be shared with…

|  | Very unlikely | Somewhat unlikely | Neither likely nor unlikely | Somewhat likely | Very likely |
| --- | --- | --- | --- | --- | --- |
| 1. Scientists in universities and other not-for-profit organizations |  |  |  |  |  |
| 1. People living with spinal cord injuries |  |  |  |  |  |
| 1. Companies developing medical products, such as prescription drugs |  |  |  |  |  |
| 1. Doctors taking care of patients |  |  |  |  |  |
| 1. Health insurance companies |  |  |  |  |  |
| 1. Government agencies |  |  |  |  |  |

1. How likely would you be to allow your anonymous, individual research data to be used in the following ways?

|  | Very unlikely | Somewhat unlikely | Neither likely nor unlikely | Somewhat likely | Very likely |
| --- | --- | --- | --- | --- | --- |
| 1. To help scientists check the accuracy of research results announced by other scientists or companies (by re-doing the analyses) |  |  |  |  |  |
| 1. To help people living with spinal cord injuries learn more about health problems that affect them |  |  |  |  |  |
| 1. To do research on health problems that affect my family or me |  |  |  |  |  |
| 1. To help get answers to scientific questions faster using information that others have already gathered |  |  |  |  |  |
| 1. To do research that will help others |  |  |  |  |  |
| 1. To help lawyers prove their case in lawsuits claiming that medical products are unsafe |  |  |  |  |  |
| 1. To learn more about diseases that only a small number of people have (by combining data from many research studies) |  |  |  |  |  |

1. Generally speaking, how often can you trust other people?
   1. Always
   2. Most of the time
   3. About half the time
   4. Once in awhile
   5. Never
2. How much do you trust…

|  | Not at all | A little | A moderate amount | A lot | A great deal |
| --- | --- | --- | --- | --- | --- |
| 1. Scientists in universities and other not-for-profit organizations |  |  |  |  |  |
| 1. People living with spinal cord injuries |  |  |  |  |  |
| 1. Companies developing medical products, such as prescription drugs |  |  |  |  |  |
| 1. Doctors taking care of patients |  |  |  |  |  |
| 1. Health insurance companies |  |  |  |  |  |
| 1. Government agencies |  |  |  |  |  |

1. Which of the following best describes how you would feel about being asked for permission to share your anonymous, individual research data with people outside of the research study you participated in (assuming your identity is well protected)? (CHECK ONE)
   1. I should be formally asked for permission to share my data, separate from my decision to be involved in the research study. My permission would broadly cover any potential use of my data in the future by others.
   2. I should be formally asked for permission to share my data, separate from my decision to be involved in the research study, every time my data is accessed by others.
   3. I do not need to provide any permission for others to access my data beyond consenting to participate in the original study.
   4. I do not want my data shared with people outside of the research study.
2. Suppose a research study has already finished and the participants weren’t informed that their data might be shared. The research team only promised their participants that they would protect against security breaches. There’s no way to get in touch with participants now. Which of the following better describes your view? (CHECK ONE)
   1. The data shouldn’t be shared.
   2. It’s okay to share the data as long as it is anonymous and there is no identifying information attached, such as my name or birthdate.
3. What is the most important reason, if any, to ask participants before sharing their anonymous, individual research data? (CHECK ONE)
   1. There is always some risk to participants, even with good security protections in place.
   2. It’s part of showing respect for participants.
   3. Neither; it’s not necessary to consult participants.
   4. Other (please write):
4. Generally speaking, if your anonymous, individual research data were shared, do you believe that you should receive monetary reimbursement? This payment would be separate from what you may have received for the original participation in the research study (if any was provided).
   1. Yes
   2. No

| Data Sharing Systems  Please read the following descriptions of 3 possible systems for sharing anonymous, individual research data. You will then be asked which system you would prefer, if you had to choose. Again, assume that **the data are anonymous**.  In the **Sponsor System,** the company or other sponsor that paid for the research holds the data and considers requests to share it. The sponsor says it will share the data whenever someone proposes a use that may advance scientific knowledge and agrees to follow data security procedures.  In the **Independent System**, an independent organization (such as a university or other not-for-profit organization) receives the data, sets up a website where people can request it, and considers requests. The organization says it will share the data whenever someone proposes a use that may advance scientific knowledge and agrees to follow data security procedures.  In the **Open Access System**, the data are posted on a website and anyone can download the data after providing their name and organization. |
| --- |

1. All things considered, which system for research data sharing would you prefer?
   1. Sponsor System
   2. Independent System
   3. Open Access System
   4. None of the above
2. In choosing that system, how important were the following to you?

|  | Not important | Somewhat unimportant | Neither important or unimportant | Somewhat important | Very important |
| --- | --- | --- | --- | --- | --- |
| a. Making sure that fair decisions are made about who gets to have the data. |  |  |  |  |  |
| b. Making sure that the data are used for legitimate purposes. |  |  |  |  |  |
| c. Having a trustworthy system. |  |  |  |  |  |
| d. Making sure the system provides good data security. |  |  |  |  |  |
| e. Making sure that the rules of the system are followed. |  |  |  |  |  |
| f. Other (please write): |  |  |  |  |  |

1. In order for you to be comfortable having your data openly shared, how certain (from 0 to 100%) do you have to be that your identity will not be revealed?
2. Overall, how do you think the potential benefits of sharing anonymous, individual research data weigh against the potential negatives? (CHECK ONE)
   1. Negatives strongly outweigh the benefits
   2. Negatives moderately outweigh the benefits
   3. Negatives outweigh the benefits a little
   4. Benefits and negatives are equal
   5. Benefits outweigh the negatives a little
   6. Benefits moderately outweigh the negatives
   7. Benefits strongly outweigh the negatives
3. Have your opinions on data sharing been altered by the COVID-19 pandemic?
   1. I am more willing to share my data
   2. It did not change my opinions
   3. I am less willing to share my data
   4. Unsure
4. If you knew that the characteristics of your SCI made your risk of re-identification higher (e.g., rare form of SCI), would that change your level of support for data sharing?
   1. Yes
   2. No
   3. Unsure

| If there is anything you would like to explain about your overall view, please do so: |
| --- |

# **SPINAL CORD INJURY SURVEY**

The following questions ask about you, your SCI, and your overall health, including what kind of SCI you have and how it affects your life. If you are uncomfortable or unable to answer a question, please feel free to skip the question or stop the survey at any time.

1. Where do you currently reside?
   1. United States
   2. Canada
   3. Other (please write):______________
2. What gender do you identify with?
   1. Female
   2. Male
   3. Non-binary/third gender
   4. Prefer to self-describe:
   5. Prefer not to answer
3. What is the highest level of formal education you have completed? (CHECK ONE)
   1. Less than high school
   2. High school diploma
   3. Diploma or certificate from trade, technical, or vocational school, or college or CEGEP
   4. Bachelor or undergraduate degree, or teacher’s college
   5. Graduate degree
   6. Prefer not to answer
4. Which of the following ethnic groups do you identify with? (CHECK ALL THAT APPLY)
   1. White
   2. Chinese
   3. First Nations, Métis, Inuit, American Indian or Alaskan Native
   4. South Asian (e.g. East Indian, Pakistani, Sri Lankan, etc.)
   5. Black or African American
   6. Latin American
   7. Southeast Asian (e.g. Vietnamese, Cambodian, Malaysian, Laotian, etc. )
   8. Arab
   9. West Asian (e.g. Iranian, Afghan, etc.)
   10. Korean
   11. Japanese
   12. Native Hawaiian or other Pacific Islander
   13. Other (please write):
   14. Don’t know
   15. Prefer not to answer
5. What is your current age (years)?

# **SPINAL CORD INJURY CLASSIFICATION**

| This section asks about your spinal cord injury and complications stemming from the injury. |
| --- |

1. What age were you when your spinal cord injury occurred (years)?
2. How many years have you been living with a spinal cord injury?
   1. Less than a year
   2. 1 year
   3. 2 years
   4. 3 years
   5. 4 years
   6. 5+ years
3. Which of the following best describes where your spinal cord injury occurred? (CHECK ONE)
4. Injury in my neck (i.e. cervical spine)
5. Injury in my upper back (i.e. upper thoracic spine)
6. Injury in my mid back (i.e. lower thoracic spine)
7. Injury in my lower back (i.e. lumbar spine)
8. Which of the following best describes the classification of your spinal cord injury? (CHECK ONE)
9. Quadriplegia (also referred to as Tetraplegia) – an injury that has affected your arms, hands, trunk and legs
10. Paraplegia – an injury that has affected your trunk and legs only (includes cauda equina)
11. Which of the following best describes the cause of your spinal cord injury? (CHECK ONE)
12. Traumatic – vehicle accident, fall, assault, sports, etc.
13. Non-traumatic – tumour, infection, congenital, neurological syndrome, degenerative spine, stroke within the spinal cord (and not in the brain), surgical complication, etc.
14. Think about your day-to-day mobility. To get around, do you usually walk or use a wheelchair? Which of the following statements best fits your main way of getting around? If you use more than one way, equally, check both ways.
    1. I walk without the help of a special aide, tool, or person
    2. I walk with the help of a special aide, tool, or person
    3. I use a manual wheelchair
    4. I use a power wheelchair or scooter
    5. Other: _______
15. Sometimes a spinal cord injury causes issues secondary to the actual injury. How often have you experienced the following problems in the past 4 weeks?

|  | Never | Occasionally | Sometimes | Most of the time | All of the time |
| --- | --- | --- | --- | --- | --- |
| 1. Sexual dysfunction |  |  |  |  |  |
| 1. Pain |  |  |  |  |  |
| 1. Urinary tract infections |  |  |  |  |  |
| 1. Joint contractures (one or more joints that are frozen and do not move) |  |  |  |  |  |
| 1. Shoulder problems |  |  |  |  |  |
| 1. Bowel or urinary incontinence |  |  |  |  |  |
| 1. Weight problems |  |  |  |  |  |
| 1. Trouble sleeping |  |  |  |  |  |
| 1. Elbow or wrist problems |  |  |  |  |  |
| 1. Pressure ulcers |  |  |  |  |  |
| 1. Fatigue |  |  |  |  |  |
| 1. Injuries that are due to a loss of sensation in that body part |  |  |  |  |  |
| 1. Lightheadedness or dizziness |  |  |  |  |  |
| 1. Constipation |  |  |  |  |  |
| 1. Respiratory infections |  |  |  |  |  |
| 1. Kidney or bladder stones |  |  |  |  |  |
| 1. Autonomic dysreflexia (sudden high blood pressure) |  |  |  |  |  |
| 1. Blood clots |  |  |  |  |  |

1. In general, would you say your health is: (CHECK ONE)
   1. Excellent
   2. Very good
   3. Good
   4. Fair
   5. Poor
2. Will you allow us to share your anonymous data from this survey with other researchers? If you answer no, we will NOT share any of your answers. If you answer yes, we will only share your **de-identified** and **anonymous** data with other researchers, your email address will NOT be shared.
   1. Yes
   2. No

| End |
| --- |

Thank you for taking the time to complete this survey!

If you would like to receive a $10 gift card, please reply the email that sent you your unique survey link indicating that you have completed the survey, and specify WHICH gift card you would like AND which country you resides in (Canada pr USA). The options are:

Starbucks or

Amazon

If you would like the giftcard to be sent to a different email, please specify that in your message.
