# Supplementary material for: Perspectives on Data Sharing in Persons With Spinal Cord Injury
Source: Neurotrauma Rep. 2023 Nov 9;4(1):781–9. doi: 10.1089/neur.2023.0035 (PMC10659015; doi:10.1089/neur.2023.0035)
Supplement: Supplemental data [file Suppl_Material.zip › French SCI Data Share Survey.docx]

Bienvenue à la

Opinion sur le partage de données par les participants avec les lésions médullaires

Conduit par:

The Kramer Lab, ICORD

L’Université de la Colombie-Britannique, Vancouver, C.-B.

En partenariat avec: North American Spinal Cord Injury Consortium (NASCIC)

Investigateur Principal: Dr. John Kramer

International Collaboration on Repair Discoveries (ICORD)

818 West 10th Avenue

Vancouver, BC V5Z 1M9

John.kramer@ubc.ca


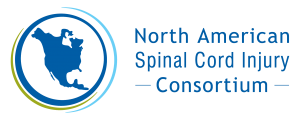

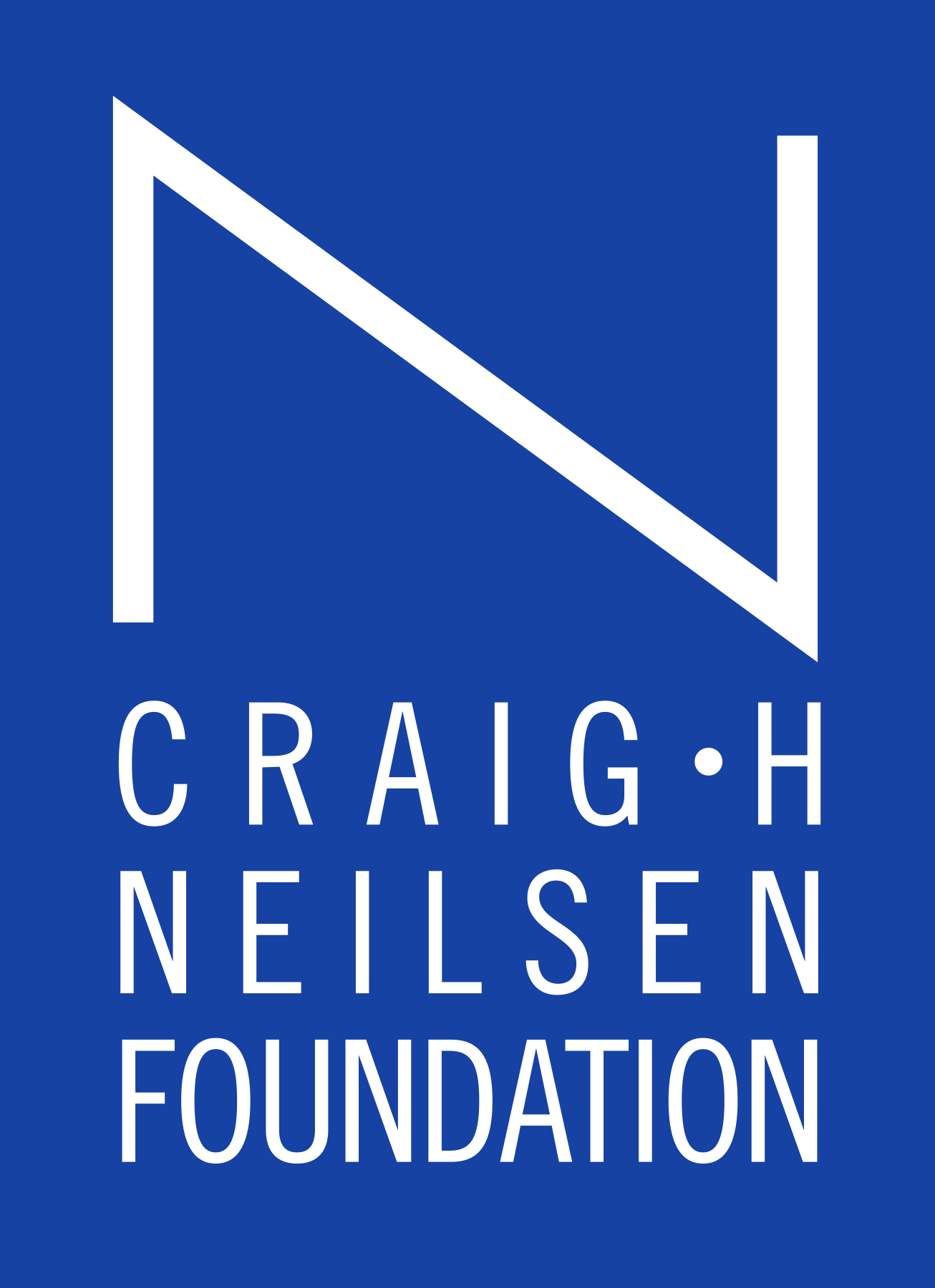


**Pourquoi faisons-nous ce questionnaire?**

Vous avez été invité à participer à une étude de recherche sur la façon dont les personnes avec des lésion médullaire ressentent les chercheurs utilisant leurs données. Plus précisément, nous souhaitons en savoir plus à propos de vos perceptions concernant **le partage de données.**

**Le partage de données** se produit lorsque les données collectées dans une étude de recherche sont partagées de manière **anonyme** avec d'autres. Cela signifie qu'un chercheur permet à d'autres de voir et d'utiliser les données individuelles qu'ils ont collectées dans une étude de recherche, après avoir supprimé les informations qui pourraient identifier des personnes (comme votre nom et votre date de naissance exacte). Cependant, d'autres informations personnelles telles que votre tranche d'âge ou des informations médicales peuvent être partagés.

Nous voulons savoir ce que vous pensez du partage de données. C’est vos informations qui seraient partagées, et nous souhaitons connaître votre opinion sur ce sujet. Nous espérons que les résultats de cette étude aideront à prendre des décisions sur l'opportunité de partager les données et les meilleures méthodes pour le faire. Que vous ayez participé à une étude de recherche ou que vous le ferez à l'avenir, votre opinion est importante pour nous. Mais sachez que votre participation à ce questionnaire et vos réponses à le questionnaire n'affecteront pas ce qu'il adviendra de vos propres données de recherche.

**En quoi consiste ce questionnaire?**

Si vous décidez de participer, vous allez remplir le questionnaire suivant qui devrait prendre environ 30 minutes. Il pose des questions sur votre santé et votre lésion médullaire, votre opinion sur les avantages et les risques potentiels du partage de données, vos préférences en matière de consentement et de protection de la vie privée, et votre niveau de confiance envers les chercheurs et autres. Nous recueillons des informations sur votre santé et vos blessures afin de connaître les caractéristiques des personnes qui ont répondu à ce questionnaire.

Vous êtes libre d'ignorer toutes les questions auxquelles vous ne souhaitez pas répondre et vous pouvez arrêter le questionnaire à tout moment. Les données recueillies dans ce questionnaire seront stockées dans un fichier crypté protégé par mot de passe sur les serveurs sécure d’UBC. Si vous décidez de partager votre courriel, cette information serais conservée séparément de votre questionnaire dans un fichier crypté protégé par mot de passe sur les serveurs sécure de l’UBC. Cela signifie qu'une fois que vous aurez soumis votre questionnaire, vous ne pourrez pas retirer vos données, car aucune information d'identification ne sera attachée. Si vous partagez votre courriel avec nous et souhaitez connaître les résultats de la recherche, nous vous fournirons un résumé des conclusions de l’étude.

**Risques et avantages**

Un avantage de participer à cette étude est la possibilité d'ajouter votre voix au débat sur le partage de données et potentiellement d'informer les futures décisions de partage de données. Nous offrons également une carte-cadeau de $10 (CAD) pour la participation. Les résultats des questionnaires sont totalement anonymes.

**Tes droits:**

Si vous avez décidé de participer à ce projet, veuillez comprendre:

- Votre participation est volontaire et vous pouvez retirer votre consentement ou arrêter de participer à tout moment sans pénalité.
- Votre capacité à continuer de participer à la recherche (si vous êtes actuellement un participant) ne sera pas affectée par votre participation à cette étude.
- Les résultats de cette étude peuvent être présentés lors de réunions scientifiques ou publiés dans des revues scientifiques, et les données du questionnaire peuvent être partagées avec d'autres chercheurs. Cependant, les informations d'identification ne seront pas divulguées.

**Si vous avez des questions:**

Si vous avez des préoccupations ou des plaintes concernant vos droits en tant que participant à la recherche et / ou vos expériences en participant à cette étude, contactez la ligne de réclamation des participants à la recherche du Bureau d'éthique de la recherche de l'Université de la Colombie-Britannique par courriel à RSIL@ors.ubc.ca ou par téléphone au 604-822-8598 (sans frais: 1-877-822-8598).

| **Déclaration de consentement :** |
| --- |
| **En remplissant et en retournant le questionnaire, vous donnez votre consentement pour participer à l'étude.** |

**Opinion sur le partage de données par les participants avec les lésions médullaires**

1. Qui répond à ce questionnaire?
   1. Je réponds ce questionnaire pour moi-même
   2. Je réponds à ce questionnaire en tant que parent ou tuteur au nom d'un enfant
   3. Je réponds à ce questionnaire au nom d'un autre adulte
   4. Autre: _____

Si vous répondez à ce questionnaire au nom d'une autre personne, veuillez répondre à ces questions à leur sujet. Si vous remplissez ce questionnaire pour vous-même, veuillez y répondre à votre sujet.

1. Avez-vous une lésion médullaire?
   1. Oui
   2. Non

Nous aimerions vous poser des questions sur votre participation la plus récente à une étude de recherche, qui aurait pu avoir lieu dans une université, un collège, un hôpital, une clinique, à votre domicile ou dans la communauté.

1. Avez - vous participé à la recherche avant de ce questionnaire ?
   1. Oui
   2. Non (passez à la question 6)

1. En pensant à la plus récente recherche étude que vous participiez,

lequel était la raison la plus importante  pour laquelle vous avez décidé d' être dans l' étude? (CHOISISSEZ-EN UN)

- 1. Je pensais qu'il y avait une chance que je puisse obtenir un avantage pour ma santé
  2. Je voulais aider les autres
  3. J'appréciais la chance de faire de l’argent
  4. Une autre raison (veuillez écrire):

1. En général, comment voulez - vous décrire votre expérience en tant que participant des recherche études?
   1. Très positif
   2. Un peu positif
   3. Ni positif ni négatif
   4. Un peu négatif
   5. Très négatif

| Nous sommes aussi intéressés par votre avis sur **le partage des données de recherche**.  Que voulons - nous dire par ça?  Le **partage de données** fait référence au partage de données **anonymes** d'études de recherche avec d'autres personnes qui ne faisaient pas partie de la recherche originale. Ces données ont tous les identifiants personnels supprimés (tels que les noms ou les dates de naissance exactes) avant d'être partagées. Les personnes qui pourraient accéder à ces données pourraient inclure d'autres chercheurs, des entreprises développant des produits médicaux, des patients, des médecins, des représentants du gouvernement, et d'autres.    Le partage des données se réfère à partager anonymement des informations sur chaque  participant de recherche, non seulement les résultats de l' étude. Ceci veut dire que toutes les informations recueillies à partir de vous dans une étude (par exemple, votre âge  gamme, type de lésion, le niveau du lésion, et votre résultat de l' étude) serait être partagée. Cela pourrait également inclure des informations génétiques. |
| --- |

1. Certaines personnes craignent de partager des données de recherche individuelles

et anonymes. Énumérés ci-dessous sont quelques-unes de ces préoccupations, à quel niveau vous inquiétez-vous au sujet de ces questions ? (SUITE À LA PAGE SUIVANTE)

|  | Pas du tout inquiet | Pas très inquiet | Un peu inquiet | Très inquiet |
| --- | --- | --- | --- | --- |
| 1. Quelqu'un qui est bon avec les ordinateurs    pourraient identifier   les données |  |  |  |  |
| 1. Les gens pourraient   être victimes de  discrimination  si l'information était  liée à eux |  |  |  |  |
| 1. Les gens pourraient   être embarrassés si l'information était liée à eux |  |  |  |  |
| 1. Les gens peuvent   utiliser les données pour faire de la  science de mauvaise qualité |  |  |  |  |
| 1. Les informations   pourraient être  utilisées dans des  projets  scientifiques desquels les participants   n’approuver pas |  |  |  |  |
| 1. Une personne ou une entreprise   pourrait gagner   beaucoup d’argent en développant des produits  utilisant  l’informations des participants |  |  |  |  |
| 1. Il pourrait être plus difficile à obtenir les participants de recherche si elles   connaissent leurs  données seront être  partagées |  |  |  |  |
| 1. Les informations   peuvent être volées |  |  |  |  |
| 1. Les entreprises   peuvent utiliser les informations à des fins de commercialisation  plutôt qu'à des fins  scientifiques |  |  |  |  |
| 1. Les scientifiques ou les entreprises pourraient injustement profiter du travail d’ autrui |  |  |  |  |
| 1. Les scientifiques et les entreprises   pourraient être moins incités à investir du temps et de l'argent dans la réalisation d' études de recherche. |  |  |  |  |
| 1. Autre préoccupation    (veuillez écrire): |  |  |  |  |

1. De cette liste des potentiels préoccupations en ce qui concerne le partage de données anonyme et individuel, laquelle et la plus importante? S'il vous plaît encercler il à la question 4 ci-dessus.
2. Bien que le partage de données de recherche individuelles anonymes suscite des inquiétudes, il existe plusieurs avantages potentiels. Dans quelle mesure pensez-vous que le partage de données de recherche individuelles et anonymes pourrait entraîner ces avantages?

|  | Pas du tout | Un peu | Modérément | Beaucoup | Énormément |
| --- | --- | --- | --- | --- | --- |
| 1. Peut aider à obtenir des réponses à des questions scienti-fiques plus rapidement en utilisant des informations que d' autres   ont déjà recueillies . |  |  |  |  |  |
| 1. Peut aider à dépenser les dollars de recherche le plus judicieusement possible. |  |  |  |  |  |
| 1. Peut réduire le coût de dévelop-pement de nouveaux produits    médicaux . |  |  |  |  |  |
| 1. Peut aider les personnes vivant avec les lésion médullaires apprendre plus sur la santé des problèmes qui les   affectent. |  |  |  |  |  |
| 1. Peut aider les scientifiques à vérifier l' exactitude des résultats de   recherche annoncés par d' autres  scientifiques ou entreprises (en   refaisant les analyses). |  |  |  |  |  |
| 1. Peut soutenir l' apprentissage   des maladies que seul un petit  nombre de personnes ont (en combinant les données de  nombreuses études de recherche ). |  |  |  |  |  |
| 1. Peut décourager les scientifiques et les entreprises de cacher ou de fausser leurs résultats (en rendant   ce possible pour les autres de vérifier leurs analyses). |  |  |  |  |  |
| 1. Peut aider les avocats à prouver leur cas dans des poursuites judiciaires   affirmant que les produits médicaux ne sont pas sûrs . |  |  |  |  |  |
| 1. Peut faire en sorte que la participation des gens aux études de recherche aboutisse au  le plus bénéfice possible. |  |  |  |  |  |
| 1. Autre avantage ( veuillez écrire ): |  |  |  |  |  |

1. Dans cette liste des avantages potentiels du partage de données de recherche individuelles et anonymes, quel est le bénéfice le plus important? Veuillez l'entourer à la question 6 ci-dessus.
2. Parmi les informations personnelles suivantes, lesquelles seriez-vous à l'aise d'être partagées en tant que données de recherche individuelles et anonymes? (SUITE À LA PAGE SUIVANTE)

|  | Très  confortable | Plutôt  confortable | Neutre | Un peu  inconfortable | Très  inconfortable | N'est pas applicable |
| --- | --- | --- | --- | --- | --- | --- |
| 1. Détails sur votre   lésion médullaire (par exemple: La façon dont il a eu lieu) |  |  |  |  |  |  |
| 1. Capacité de locomotion (par exemple: Marche) |  |  |  |  |  |  |
| 1. Santé émotionnelle (par exemple: Bonheur ,   dépression ) |  |  |  |  |  |  |
| 1. Dysfonction   sexuelle |  |  |  |  |  |  |
| 1. Spasticité   (mouvements  musculaires  spontanés et  incontrôlés ,  saccadés, tels que  contraction  musculaire  incontrôlée ou spasme ) |  |  |  |  |  |  |
| 1. Douleur |  |  |  |  |  |  |
| 1. Intestin/vessie   préoccupations (par exemple: L' incontinence, des reins pierres,  infections urinaires, la constipation ) |  |  |  |  |  |  |
| 1. Contractures articulaires (une ou plusieurs articulations qui   sont congelés ou ont limité l' amplitude de mouvement ) |  |  |  |  |  |  |
| 1. Problèmes d' épaule , de coude ou de poignet (par exemple: Douleur dans ces   articulations) |  |  |  |  |  |  |
| 1. Maladie   métabolique (par exemple: Diabète ) |  |  |  |  |  |  |
| 1. Problèmes de poids |  |  |  |  |  |  |
| 1. Problèmes cardiaques ou   sanguins (par exemple: pression artérielle élevé, dysréflexie autonome, caillots ) |  |  |  |  |  |  |
| 1. Troubles du sommeil |  |  |  |  |  |  |
| 1. Problèmes   respiratoires (par exemple: Pneumonie) |  |  |  |  |  |  |
| 1. Escarres |  |  |  |  |  |  |
| 1. Problèmes avec le fonction cérébrale (par exemple: Parler , la compréhension des mots , mémoire, vision) |  |  |  |  |  |  |
| 1. Fatigue |  |  |  |  |  |  |
| 1. Les blessures qui sont dues à une perte de sensation dans cette partie du corps |  |  |  |  |  |  |
| 1. Étourdissements |  |  |  |  |  |  |
| 1. Autre   préoccupation  (veuillez écrire ) : |  |  |  |  |  |  |

1. Dans quelle mesure pensez-vous que les groupes suivants pourraient bénéficier du partage de données de recherche individuelles et anonymes?

|  | Pas du tout | Un peu | Une quantité modérée | Beaucoup | Énormément |
| --- | --- | --- | --- | --- | --- |
| 1. Scientifiques dans les universités et autres organisations à but non lucratif |  |  |  |  |  |
| 1. Les personnes avec des lésions médullaires |  |  |  |  |  |
| 1. Entreprises développant des produits médicaux, tels que des médicaments sur ordonnance |  |  |  |  |  |
| 1. Médecins prenant soin des patients |  |  |  |  |  |
| 1. Compagnie d'assurance maladie |  |  |  |  |  |
| 1. Organismes gouvernemen-taux |  |  |  |  |  |

1. Quelle est la probabilité que vous autorisiez le partage de vos données de recherche individuelles et anonymes avec…

|  | Très peu  probable | Un peu probable | Ni probable n’ou  peu probable | Plutôt  probable | Très  probable |
| --- | --- | --- | --- | --- | --- |
| 1. Scientifiques dans les universités et autres organisations à but non lucratif |  |  |  |  |  |
| 1. Les personnes avec des lésions médullaires |  |  |  |  |  |
| 1. Entreprises développant des produits médicaux, tels que des médicaments sur ordonnance |  |  |  |  |  |
| 1. Médecins prenant soin des patients |  |  |  |  |  |
| 1. Compagnie d'assurance maladie |  |  |  |  |  |
| 1. Organismes gouvernemen-taux |  |  |  |  |  |

1. Dans quelle mesure seriez-vous susceptible d'autoriser l'utilisation de vos données de recherche individuelles et anonymes de la manière suivante?

|  | Très peu  probable | Un peu  probable | Ni probable n’ou  peu probable | Plutôt  probable | Très  probable |
| --- | --- | --- | --- | --- | --- |
| 1. Aider les scientifiques à vérifier l' exactitude des résultats de    recherche annoncés par d' autres scientifiques ou  entreprises (en refaisant les analyses) |  |  |  |  |  |
| 1. Pour aider les personnes vivant avec les lésions médullaires apprendre plus sur leurs  problèmes de santé |  |  |  |  |  |
| 1. Faire des recherches sur les   problèmes de santé qui affectent   ma famille ou moi |  |  |  |  |  |
| 1. Pour aider à obtenir des réponses aux questions   scientifiques plus rapidement en utilisant les informations que d' autres ont déjà recueillies |  |  |  |  |  |
| 1. Pour ce faire la recherche qui va aider les autres |  |  |  |  |  |
| 1. Aider les avocats à prouver leur cause dans des poursuites judiciaires   affirmant que les produits   médicaux ne sont pas sûrs |  |  |  |  |  |
| 1. Pour en savoir plus sur les maladies que seul   un petit nombre de personnes ont (en combinant les données de nombreuses   études de recherche ) |  |  |  |  |  |

1. En général, à quelle fréquence pouvez-vous faire confiance à d'autres personnes?
   1. Toujours
   2. La plupart du temps
   3. Environ la moitié du temps
   4. De temps en temps
   5. Jamais
2. À quel point faites-vous confiance…

|  | Pas du tout | Un peu | Modérément | Beaucoup | Énormément |
| --- | --- | --- | --- | --- | --- |
| 1. Scientifiques dans les universités et autres organisations à but non lucratif |  |  |  |  |  |
| 1. Les personnes avec des lésions médullaires |  |  |  |  |  |
| 1. Entreprises développant des produits médicaux, tels que des médicaments sur ordonnance |  |  |  |  |  |
| 1. Médecins prenant soin des patients |  |  |  |  |  |
| 1. Compagnie d'assurance maladie |  |  |  |  |  |
| 1. Organismes gouvernementaux |  |  |  |  |  |

1. Lequel des énoncés suivants décrit le mieux ce que vous ressentiriez lorsqu'on vous demande la permission de partager vos données de recherche individuelles et anonymes avec des personnes extérieures à l'étude de recherche à laquelle vous avez participé (en supposant que votre identité est bien protégée)? (CHOISISSEZ-EN UN)
   1. Je devrais être formellement demandé la permission de partager mes données, séparer de ma décision de participer à l'étude de recherche. Ma permission couvrirait largement toute utilisation potentielle de mes données à l'avenir par d'autres.
   2. Je devrais être formellement demandé la permission de partager mes données, séparées de ma décision de participer à l'étude de recherche, chaque fois que mes données sont accessibles par d'autres.
   3. Je n'ai pas besoin de donner la permission à d'autres d'accéder à mes données au-delà du consentement à participer à l'étude originale.
   4. Je ne veux pas que mes données soient partagées avec des personnes en dehors de l'étude de recherche.
2. Supposons qu’une étude de recherche soit déjà terminée et que les participants ne soient pas informés que leurs données pourraient être partagées. L'équipe de recherche a seulement promis à ses participants de se protéger contre les failles de sécurité. Il n'y a aucun moyen d'entrer en contact avec les participants maintenant. Lequel des énoncés suivants décrit le mieux votre point de vue? (CHOISISSEZ-EN UN)
   1. Les données ne doivent pas être partagées.
   2. Vous pouvez partager les données tant qu'elles sont anonymes et qu'aucune information d'identification n'est jointe, comme mon nom ou ma date de naissance.
3. Quelle est la raison la plus importante, le cas échéant, de demander aux participants avant de partager leurs données de recherche individuelles et anonymes? (CHOISISSEZ-EN UN)
   1. Il y a toujours un risque pour les participants, même avec de bonnes protections de sécurité en place.
   2. Cela fait partie du respect des participants.
   3. Ni; il n’est pas nécessaire de consulter les participants.
   4. Autre (veuillez écrire):
4. En générale, si vos données de recherche individuelles et anonymes étaient partagées, pensez-vous que vous devriez recevoir un remboursement monétaire? Ce paiement serait distinct de ce que vous pourriez avoir reçu pour la participation initiale à l'étude de recherche (le cas échéant).
   1. Oui
   2. Non

| Systèmes de partage de données  Veuillez lire les descriptions suivantes de 3 systèmes possibles pour partager des données de recherche individuelles et anonymes. On vous demandera alors quel système vous préférez, si vous deviez choisir. Encore une fois, supposons que les **données sont anonymes**.  Dans le **système de parrainage,** la société ou sponsor qui a payé la recherche détient les données et prend en compte les demandes de partage. Le sponsor dit qu'il partagera les données chaque fois que quelqu'un proposera une utilisation susceptible de faire progresser les connaissances scientifiques et accepte de suivre les procédures de sécurité des données.  Dans le **système indépendant**, une organisation indépendante (telle qu'une université ou une autre organisation à but non lucratif) reçoit les données, crée un site Web sur lequel les gens peuvent en faire la demande et examine les demandes. L'organisation dit qu'elle partagera les données chaque fois que quelqu'un proposera une utilisation susceptible de faire progresser les connaissances scientifiques et accepte de suivre les procédures de sécurité des données.  Dans le **système Open Access**, les données sont publiées sur un site Web et n'importe qui peut télécharger les données après avoir fourni son nom et son organisation. |
| --- |

1. Tout bien considéré, quel système de partage des données de recherche préférez-vous?
   1. Système de parrainage
   2. Système indépendant
   3. Système d'accès ouvert
   4. Aucune de ces réponses
2. En choisissant ce système, dans quelle mesure les éléments suivants ont-ils été importants pour vous?

|  | Pas important | Un peu sans   importance | Ni important ni sans importance | Assez  important | Très important |
| --- | --- | --- | --- | --- | --- |
| a. Faire assurer que  justes décisions sont prises au sujet qui  obtient  les données. |  |  |  |  |  |
| b. Faire assurer que les données sont utilisées  pour les fins légitimes. |  |  |  |  |  |
| c. Avoir un système  fiable . |  |  |  |  |  |
| d. S'assurer que le système offre une bonne sécurité des données . |  |  |  |  |  |
| e. Faire assurer que les règles du système sont respectées . |  |  |  |  |  |
| f. Autre (veuillez écrire ): |  |  |  |  |  |

1. Pour que vous soyez à l'aise avec le partage ouvert de vos données, dans quelle mesure devez-vous être certain (de 0 à 100%) que votre identité ne sera pas révélée?
2. Dans l'ensemble, comment pensez-vous que les avantages potentiels du partage de données de recherche individuelles anonymes se comparent aux inconvénients potentiels? (CHOISISSEZ-EN UN)
   1. Les inconvénients l'emportent largement sur les avantages
   2. Les inconvénients l'emportent modérément sur les avantages
   3. Les inconvénients l'emportent un peu sur les avantages
   4. Les avantages et les inconvénients sont égaux
   5. Les avantages l'emportent un peu sur les inconvénients
   6. Les avantages l'emportent modérément sur les inconvénients
   7. Les avantages l'emportent largement sur les inconvénients
3. Votre opinion sur le partage de données a-t-elle été modifiée par la pandémie COVID-19?
   1. Je suis plus disposé à partager mes données
   2. Cela n'a pas changé mes opinions
   3. Je suis moins disposé à partager mes données
   4. Incertain
4. Si vous saviez que les caractéristiques de votre lésion médullaire augmentaient votre risque de ré-identification (par exemple, une forme rare de lésion), cela changerait-il votre niveau de soutien pour le partage des données?
   1. Oui
   2. Non
   3. Incertain

| Si vous souhaitez expliquer quelque chose sur votre vue d'ensemble, veuillez le faire: |
| --- |

# **QUESTIONNAIRE SUR LES LÉSION MÉDULLAIRES**

Les questions suivantes portent sur vous, votre lésion médullaire et votre état de santé général, y compris le type de votre lésion médullaire que vous avez et comment cela affecte votre vie. Si vous êtes mal à l'aise ou incapable de répondre à une question, n'hésitez pas à sauter la question ou à arrêter le questionnaire à tout moment.

1. Où habitez-vous actuellement?
   1. Les États Unis
   2. Canada
   3. Autre (veuillez écrire):______________
2. À quel sexe vous identifiez-vous?
   1. Femme
   2. Mâle
   3. Non binaire/troisième sexe
   4. Préférez vous décrire:
   5. Préfère ne pas répondre
3. Quel est le niveau d'éducation formelle le plus élevé que vous ayez atteint? (CHOISISSEZ-EN UN)
   1. Moins que le secondaire
   2. Diplôme d’études secondaires
   3. Diplôme ou certificat d'une école professionnelle, technique ou professionnelle, d'un collège ou d'un cégep
   4. Baccalauréat, ou collège enseignant
   5. Diplôme d'études supérieures
   6. Préfère ne pas répondre
4. Avec lequel des groupes ethniques suivants vous identifiez-vous? (CHOISISSEZ TOUT CE QUI S'APPLIQUE)
   1. Blanc
   2. Chinois
   3. Premières Nations, Métis, Inuits, Amérindiens ou autochtones d'Alaska
   4. Asie du Sud (par exemple, Inde orientale, pakistanaise, sri-lankaise, etc.)
   5. Noir ou afro-américain
   6. Latino-américain
   7. Asie du Sud-Est (par exemple, vietnamien, cambodgien, malais, laotien, etc.)
   8. Arabe
   9. Asie occidentale (par exemple iranien, afghan, etc.)
   10. Coréen
   11. Japonais
   12. Hawaïen indigène ou autre insulaire du Pacifique
   13. Autre (veuillez écrire):
   14. Je ne sais pas
   15. Préfère ne pas répondre
5. Quel est votre âge actuel (années)?

**CLASSIFICATION DES LÉSIONS MÉDULLARIES**

| Cette section pose des questions sur votre lésion médullaire et les complications causés par la lésion. |
| --- |

1. Quel âge aviez-vous lorsque votre lésion médullaire est survenue (années)?
2. Depuis combien d’années vivez-vous avec une lésion médullaire?
   1. Moins qu’un an
   2. 1 année
   3. 2 ans
   4. 3 ans
   5. 4 ans
   6. 5+ ans
3. Lequel des énoncés suivants décrit le mieux l'endroit où votre lésion médullaire s'est produite? (CHOISISSEZ-EN UN)
   1. Blessure au cou (c'est-à-dire à la colonne cervicale)
   2. Blessure dans le haut du dos (c'est-à-dire à la Colonne thoracique supérieure)
   3. Blessure au milieu du dos (c'est-à-dire à la Colonne thoracique inférieure)
   4. Blessure dans le bas du dos (c'est-à-dire à la Colonne lombaire)
4. Lequel des énoncés suivants décrit le mieux la classification de votre lésion médullaire? (CHOISISSEZ-EN UN)
   1. Quadriplégie (également appelée tétraplégie) - une lésion qui a affecté vos bras, mains, tronc et jambes
   2. Paraplégie - une lésion qui n'a touché que le tronc et les jambes (comprend la queue de cheval)
5. Lequel des énoncés suivants décrit le mieux la cause de votre lésion médullaire? (CHOISISSEZ-EN UN)
6. Traumatique - accident de véhicule, chute, agression, sport, etc.
7. Non traumatique - tumeur, infection, congénital, syndrome neurologique, colonne vertébrale dégénérative, accident vasculaire cérébral dans la moelle épinière (et non dans le cerveau), complication chirurgicale, etc.
8. Pensez à votre mobilité au quotidien. Pour vous déplacer, marchez-vous ou utilisez-vous habituellement un fauteuil roulant? Laquelle des affirmations suivantes correspond le mieux à votre principale façon de vous déplacer? Si vous utilisez plusieurs méthodes, également, vérifiez les deux méthodes.
   1. Je marche sans l'aide d'un assistant spécial, d'un outil ou d'une personne
   2. Je marche avec l'aide d'un assistant spécial, d'un outil ou d'une personne
   3. J'utilise un fauteuil roulant manuel
   4. J'utilise un fauteuil roulant électrique ou un scooter
   5. Autre: _______
9. Parfois, une lésion de la moelle épinière provoque des problèmes secondaires à la blessure réelle. À quelle fréquence avez-vous rencontré les problèmes suivants au cours des 4 dernières semaines?

|  | Jamais | Parfois | Quelquefois | La plupart du temps | Tout le temps |
| --- | --- | --- | --- | --- | --- |
| 1. Dysfonction sexuelle |  |  |  |  |  |
| 1. Douleur |  |  |  |  |  |
| 1. Infections des voies urinaires |  |  |  |  |  |
| 1. Contractures articulaires (une ou plusieurs articulations qui sont   gelées et ne bougent pas) |  |  |  |  |  |
| 1. Problèmes d' épaule |  |  |  |  |  |
| 1. Incontinence   intestinale ou urinaire |  |  |  |  |  |
| 1. Problèmes de poids |  |  |  |  |  |
| 1. Troubles du sommeil |  |  |  |  |  |
| 1. Problèmes de coude ou de poignet |  |  |  |  |  |
| 1. Escarres |  |  |  |  |  |
| 1. Fatigue |  |  |  |  |  |
| 1. Les blessures qui sont dues à une perte de sensation dans cette   partie du corps |  |  |  |  |  |
| 1. Étourdissements |  |  |  |  |  |
| 1. Constipation |  |  |  |  |  |
| 1. Infections respiratoires |  |  |  |  |  |
| 1. Rein ou la vessie pierres |  |  |  |  |  |
| 1. Dysréflexie Autonome  (soudaine élevée sang pression) |  |  |  |  |  |
| 1. Caillots sanguins |  |  |  |  |  |

1. En général, diriez-vous que votre santé est: (CHOISISSEZ-EN UN)
   1. Excellent
   2. Très bien
   3. Bien
   4. Comme si comme ça
   5. Pauvre
2. Voulez-vous nous permettre à partager vos données anonymes de ce questionnaire avec d'autres chercheurs? Si vous répondez non, nous ne partagerons aucune de vos réponses. Si vous répondez oui, nous partagerons seulement vos données anonymes avec d'autres chercheurs, votre adresse e-mail ne sera PAS partagée.
   1. Oui
   2. Non

| **Fin** |
| --- |

Merci d'avoir pris le temps de répondre à ce questionnaire!

Si vous souhaitez recevoir une carte-cadeau de 50 $, veuillez répondre à l'e-mail qui vous a envoyé votre lien unique pour le questionnaire indiquant que vous avez répondu à le questionnaire, et spécifiez QUELLE carte-cadeau vous souhaitez ET dans quel pays vous résidez (Canada ou États-Unis).

Starbucks,

ou Amazon.

Si vous souhaitez que la carte-cadeau soit envoyée à un autre e-mail, veuillez l'indiquer dans votre message.
