# Supplementary material for: Perspectives on Data Sharing in Persons With Spinal Cord Injury
Source: Neurotrauma Rep. 2023 Nov 9;4(1):781–9. doi: 10.1089/neur.2023.0035 (PMC10659015; doi:10.1089/neur.2023.0035)
Supplement: Supplemental data [file Suppl_Material.zip › Spanish SCI Data Share Survey.docx]

Bienvenida a la

Opiniones de los participantes con lesiones de la médula espinal de la investigación sobre la encuesta de intercambio de datos

Llevada a cabo por:

El laboratorio Kramer, ICORD

University of British Columbia, Vancouver, B.C.

En colaboración con:

Consorcio de Lesiones de la Médula Espinal de América del Norte (NASCIC)

Investigador principal: Dr. John Kramer

International Collaboration on Repair Discoveries (ICORD)

818 West 10th Avenue


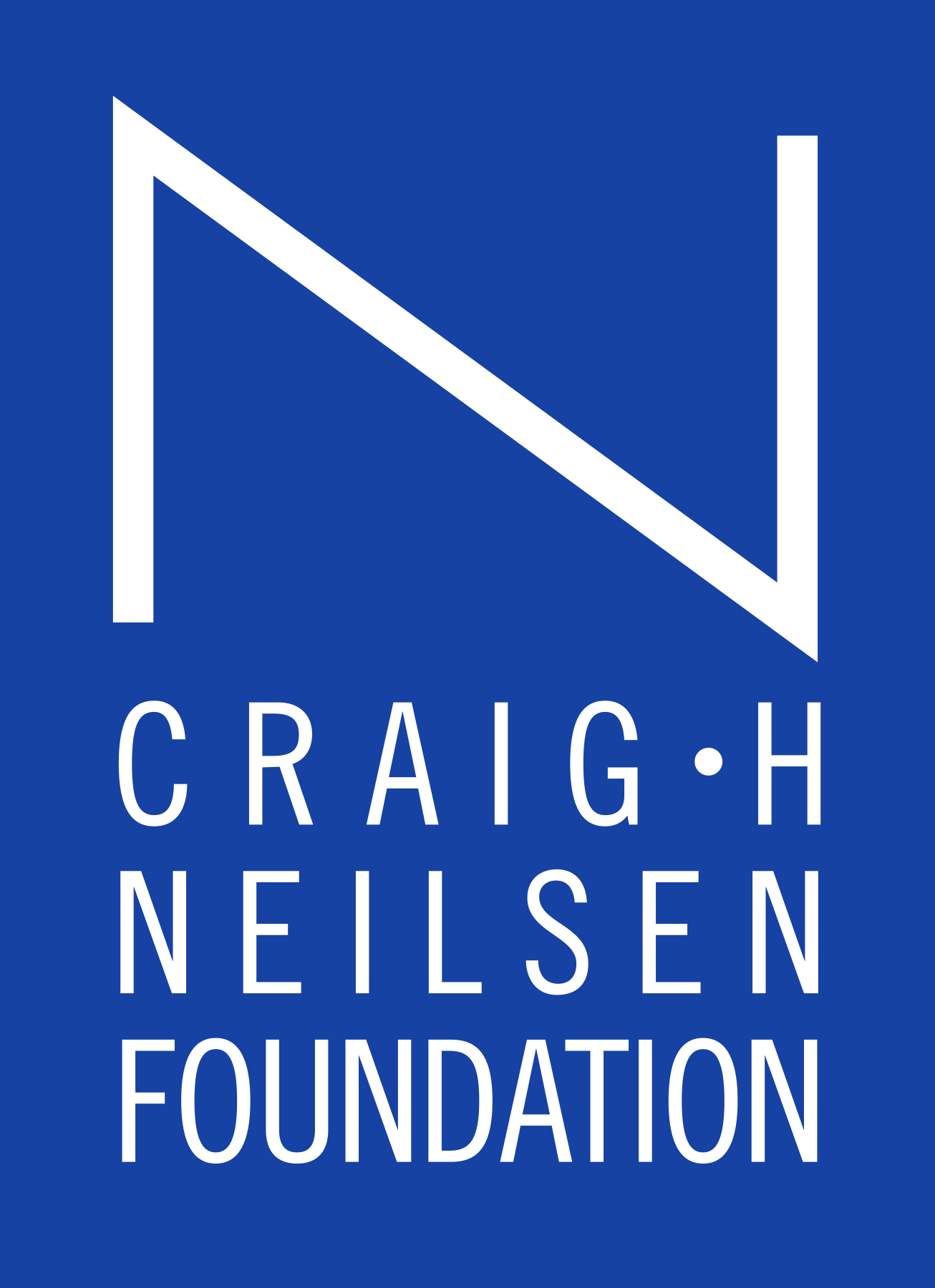
Vancouver, BC V5Z 1M9

John.kramer@ubc.ca


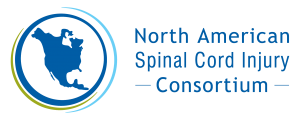


**¿Por qué estamos haciendo esta encuesta?**

Usted ha sido invitado a participar en un estudio que investiga cómo se sienten las personas con lesión de la médula espinal (LME) sobre los investigadores que usan sus datos. Específicamente, estamos interesados ​​en aprender más sus percepciones sobre ***el intercambio de datos***.

***El intercambio de datos*** es cuando los datos recopilados en un estudio de investigación se comparten **anónimamente** con otros. Esto significa que un investigador permite que otros vean y usen los datos individuales que recopilaron en un estudio de investigación, después de eliminar información que podría identificar a las personas (como su nombre y fecha de nacimiento). Sin embargo, otra información personal como su rango de edad o información médica podría ser proporcionada.

**Queremos saber cómo se siente con respecto al intercambio de datos**. En el caso de que su información sea compartida, queremos conocer su opinión sobre este tema. Es nuestro deseo que los resultados de este estudio nos ayuden a tomar decisiones sobre si los datos deben compartirse y cómo. Si ha participado en un estudio de investigación o lo hará en el futuro, su opinión es importante para nosotros. Pero tenga en cuenta que **su participación y sus respuestas en esta encuesta no afectarán lo que sucede con sus propios datos de investigación**.

**¿En qué consiste la encuesta?**

Si decide participar, se le pedirá que complete la siguiente encuesta que debería tomar aproximadamente 30 minutos. Contiene preguntas sobre su estado de salud y sobre la lesión de la médula espinal, como también su opinión sobre los posibles beneficios y riesgos del intercambio de datos. También, sus preferencias con respecto al consentimiento informado, la protección de su privacidad y su nivel de confianza en los investigadores y otros. Estamos recopilando información sobre su salud y lesión para conocer las características de las personas que respondieron esta encuesta.

Puede omitir cualquier pregunta que no desee responder y puede detener la encuesta en cualquier momento. Los datos recopilados de esta encuesta se almacenarán en un archivo cifrado protegido con contraseña en los servidores seguros de UBC. Si decide proporcionarnos su correo electrónico, esta información se mantendrá separada de su encuesta en un archivo cifrado protegido con contraseña en los servidores seguros de UBC. Esto significa que una vez que envíe su encuesta, no podrá retirar sus datos, ya que no tendrá información de identificación adjunta. Si comparte su correo electrónico con nosotros y desea conocer los resultados del estudio, le proporcionaremos un resumen de los hallazgos del estudio.

**Riesgos y beneficios**

Un beneficio por su participación en este estudio es la oportunidad de agregar su voz al debate sobre el intercambio de datos, y potencialmente informar futuras decisiones de intercambio de datos. También estamos ofreciendo una tarjeta de regalo de $10 por participar. Los resultados de las encuestas son completamente anónimos.

**Sus derechos:**

Si ha decidido participar en este proyecto, comprenda que:

- Su participación es voluntaria y puede retirar su consentimiento o dejar de participar en cualquier momento sin penalización.
- Su capacidad para continuar participando en la investigación (si actualmente es un participante) no se verá afectada por su participación en este estudio de encuesta.
- Los resultados de este estudio pueden presentarse en reuniones científicas o publicarse en revistas científicas, y los datos de la encuesta se pueden compartir con otros investigadores. Sin embargo, la información sobre identificación no será revelada.

**Si tiene preguntas:**

Si tiene alguna preocupación o queja sobre sus derechos como participante de la investigación y / o sus experiencias mientras participa en este estudio, comuníquese con la Línea de Quejas de Participantes de Investigación en la Oficina de Ética de Investigación de la Universidad de Columbia Británica por correo electrónico a RSIL@ors.ubc.ca o por teléfono al 604-822-8598 (llamada gratuita: 1-877-822-8598).

| **Declaración de consentimiento:** |
| --- |
| **Al completar y devolver la encuesta, usted está dando su consentimiento informado para participar en el estudio.** |

**Encuesta sobre el Intercambio de Datos de Estudios de La Investigación de Lesiones de la Médula Espinal**

1. ¿Quién está completando esta encuesta?
   1. Estoy completando esta encuesta por mí mismo
   2. Estoy completando esta encuesta como padre o tutor en nombre de un niño
   3. Estoy completando esta encuesta en nombre de otro adulto
   4. Otro:

Si está completando esta encuesta en nombre de otra persona, responda estas preguntas sobre ellos. Si está completando esta encuesta usted mismo, responda sobre usted.

1. ¿Tiene una lesión en la médula espinal?
   1. Sí
   2. No

Nos gustaría preguntarle sobre su **participación más reciente** en un **estudio de investigación**, que podría haberse efectuado en una universidad, colegio, hospital, clínica, en su hogar o en la comunidad.

1. ¿Ha participado en una investigación previa a esta encuesta?
   1. Sí
   2. No (pase a la pregunta 6)
2. Pensando en el estudio de investigación más reciente en el que participó, ¿cuál fue la razón más importante por la que decidió participar en el estudio? (MARCAR UNO)
   1. Pensé que había una posibilidad de obtener un beneficio para la salud.
   2. Quería ayudar a otros
   3. Valoré la oportunidad de ganar algo de dinero
   4. Alguna otra razón (por favor escriba):
3. En general, ¿cómo describiría su experiencia como participante en un estudio de investigación?
   1. Muy positivo
   2. Relativamente positivo
   3. Ni positivo ni negativo
   4. Relativamente negativo
   5. Muy negativo

También nos interesa su opinión sobre **el intercambio de datos de investigación**. ¿Qué queremos decir con esto?

***El intercambio de datos*** se refiere al intercambio de datos **anónimos** del estudio de investigación con otros que no formaron parte de la investigación original. Estos datos tienen todos los identificadores personales eliminados (como nombres o fechas de nacimiento) antes de ser compartidos. Las personas que podrían acceder a estos datos podrían incluir otros investigadores, compañías que desarrollan productos médicos, pacientes, médicos, funcionarios de gobierno y otros.

El intercambio de datos se refiere al intercambio de información anónima sobre cada participante individual de la investigación, no solo los resultados del estudio. Esto significa que toda la información recopilada de usted en un estudio (por ejemplo, su rango de edad, tipo de LME, nivel de LME y su resultado del estudio) se compartiría. Esto también podría incluir información genética.

1. A algunas personas les preocupa compartir datos de la Investigación individual anónima. A continuación, se enumeran algunas de estas preocupaciones. ¿Qué tan preocupado está usted por estos problemas? (CONTINÚA EN LA SIGUIENTE PÁGINA)

|  | Nada preocupado | No muy preocupado | Relativamente preocupado | Muy preocupado |
| --- | --- | --- | --- | --- |
| 1. Alguien que sea bueno con las computadoras podría identificar los datos. |  |  |  |  |
| 1. Las personas podrían ser discriminadas si la información se vincula a ellas |  |  |  |  |
| 1. Las personas podrían sentirse avergonzadas si la información se vincula a ellas |  |  |  |  |
| 1. La gente podría usar los datos para hacer ciencia de baja calidad |  |  |  |  |
| 1. La información se podría usar en proyectos científicos que los participantes no aprobarían |  |  |  |  |
| 1. Algunas persona o empresa podría ganar mucho dinero desarrollando productos utilizando la información de las personas |  |  |  |  |
| 1. Podría ser más difícil lograr que las personas acepten participar en estudios de investigación si saben que sus datos serán compartidos |  |  |  |  |
| 1. La información podría ser robada |  |  |  |  |
| 1. Las empresas pueden utilizar la información con fines de marketing en lugar de fines científicos. |  |  |  |  |
| 1. Los científicos o las empresas podrían injustamente "beneficiarse gratuitamente" con el trabajo de otros |  |  |  |  |
| 1. Los científicos y las empresas podrían tener menos incentivos para invertir tiempo y dinero en realizar estudios de investigación. |  |  |  |  |
| 1. Otra preocupación (por favor escriba): |  |  |  |  |

1. De esa lista de posibles preocupaciones con respecto a compartir datos de la investigación individual anónima, ¿cuál es LA MÁS IMPORTANTE? Encierre con un círculo su respuesta en la pregunta número 4 citada anteriormente.
2. Aunque existen preocupaciones acerca de compartir datos de la investigación individual anónima, también existen varios beneficios potenciales. ¿Cuánto cree que compartir datos de la investigación individual anónima podría conducir a estos beneficios potenciales?

|  | De ningún modo | Un poco | Una cantidad moderada | Bastante | Mucho |
| --- | --- | --- | --- | --- | --- |
| 1. Puede ayudar a obtener respuestas a preguntas científicas más rápido utilizando información que otros ya han reunido. |  |  |  |  |  |
| 1. Puede ayudar a garantizar que los dólares para la investigación se gasten de la manera más inteligente posible |  |  |  |  |  |
| 1. Puede reducir el costo del desarrollo de nuevos productos médicos. |  |  |  |  |  |
| 1. Puede ayudar a las personas que viven con lesiones de la médula espinal a aprender más sobre los problemas de salud que les afectan |  |  |  |  |  |
| 1. Puede ayudar a los científicos a verificar la precisión de los resultados de la investigación anunciados por otros científicos o compañías (al rehacer los análisis) |  |  |  |  |  |
| 1. Puede respaldar el aprendizaje sobre enfermedades que solo tiene un pequeño número de personas (combinando datos de muchos estudios de investigación) |  |  |  |  |  |
| 1. Puede disuadir a los científicos y las empresas de ocultar o distorsionar los resultados de sus estudios de investigación (haciendo posible que otros verifiquen sus análisis) |  |  |  |  |  |
| 1. Puede ayudar a los abogados a probar su caso en demandas que alegan que los productos médicos no son seguros |  |  |  |  |  |
| 1. Puede garantizar que la participación de las personas en estudios de investigación conduzca al mayor beneficio científico posible |  |  |  |  |  |
| 1. Otro beneficio (por favor escriba): |  |  |  |  |  |

1. De la lista de beneficios potenciales para compartir datos de la investigación individual anónima, ¿cuál es El BENEFICIO MÁS IMPORTANTE? Encierre con un círculo en la pregunta número 6 citada anteriormente.
2. ¿Cuáles de las siguiente Información Personal se sentiría cómodo compartiendo como datos de la investigación individual anónima? (CONTINÚA EN LA SIGUIENTE PÁGINA)

|  | Muy cómodo | Relativamente cómodo | Neutral | Relativamente incomodo | Muy incómodo | No aplica |
| --- | --- | --- | --- | --- | --- | --- |
| 1. Detalles sobre su lesión de la médula espinal (por ejemplo, cómo ocurrió) |  |  |  |  |  |  |
| 1. Capacidad de locomoción (por ejemplo, caminar) |  |  |  |  |  |  |
| 1. Salud emocional (por ejemplo, felicidad, depresión) |  |  |  |  |  |  |
| 1. Disfunción sexual |  |  |  |  |  |  |
| 1. Espasticidad (movimientos musculares espasmódicos incontrolados, como contracciones musculares incontroladas o espasmo) |  |  |  |  |  |  |
| 1. Dolor |  |  |  |  |  |  |
| 1. Problemas intestinales / y de vejiga (por ejemplo, incontinencia, cálculos renales, infecciones del tracto urinario, estreñimiento) |  |  |  |  |  |  |
| 1. Contractura articular (una o más articulaciones que están congeladas o tienen un rango de movimiento limitado) |  |  |  |  |  |  |
| 1. Problemas de hombro, codo o muñeca (por ejemplo, dolor en estas articulaciones) |  |  |  |  |  |  |
| 1. Enfermedad metabólica (por ejemplo, Diabetes) |  |  |  |  |  |  |
| 1. Problemas de peso |  |  |  |  |  |  |
| 1. Problemas cardíacos o sanguíneos (por ejemplo, presión arterial alta, disreflexia autónoma, coágulos) |  |  |  |  |  |  |
| 1. Problemas para dormir |  |  |  |  |  |  |
| 1. Problemas respiratorios (por ejemplo, neumonía) |  |  |  |  |  |  |
| 1. Úlceras por presión |  |  |  |  |  |  |
| 1. Problemas con la función cerebral (por ejemplo, hablar, comprender palabras, memoria, visión) |  |  |  |  |  |  |
| 1. Fatiga |  |  |  |  |  |  |
| 1. Lesiones que se deben a una pérdida de sensibilidad en cierta parte del cuerpo. |  |  |  |  |  |  |
| 1. Aturdimiento o mareos |  |  |  |  |  |  |
| 1. Otra preocupación (por favor escribe): |  |  |  |  |  |  |

1. ¿Cuánto cree que podrían beneficiarse los siguientes grupos al compartir datos de la investigación individual anónima?

|  | De ningún modo | Un poco | Una cantidad moderada | Bastante | Mucho |
| --- | --- | --- | --- | --- | --- |
| 1. Científicos en universidades y otras organizaciones sin fines de lucro |  |  |  |  |  |
| 1. Personas que viven con lesiones de la médula espinal |  |  |  |  |  |
| 1. Empresas que desarrollan productos médicos, como medicamentos recetados |  |  |  |  |  |
| 1. Doctores cuidando pacientes |  |  |  |  |  |
| 1. Compañías de seguros de salud |  |  |  |  |  |
| 1. Agencias gubernamentales |  |  |  |  |  |

1. ¿Qué posibilidades hay de permitir que sus datos de la investigación individual anónima se compartan con ...?

|  | De ningún modo | Un poco | Una cantidad moderada | Bastante | Mucho |
| --- | --- | --- | --- | --- | --- |
| 1. Científicos en universidades y otras organizaciones sin fines de lucro |  |  |  |  |  |
| 1. Personas que viven con lesiones de la médula espinal |  |  |  |  |  |
| 1. Empresas que desarrollan productos médicos, como medicamentos recetados |  |  |  |  |  |
| 1. Doctores cuidando pacientes |  |  |  |  |  |
| 1. Compañías de seguros de salud |  |  |  |  |  |
| 1. Agencias gubernamentales |  |  |  |  |  |

1. ¿Qué posibilidades hay de permitir que sus datos de la investigación individual anónima se utilicen de las siguientes maneras?

|  | Muy improbable | Algo poco probable | Ni probable ni improbable | Algo probable | Muy probable |
| --- | --- | --- | --- | --- | --- |
| 1. Para ayudar a los científicos a verificar la precisión de los resultados de la investigación anunciados por otros científicos o empresas (rehaciendo los análisis) |  |  |  |  |  |
| 1. Para ayudar a las personas que viven con lesiones de la médula espinal a aprender más sobre los problemas de salud que les afectan |  |  |  |  |  |
| 1. Investigar problemas de salud que nos afectan a mi familia o a mí |  |  |  |  |  |
| 1. Para ayudar a obtener rápidamente respuestas a preguntas científicas utilizando información que otros ya han reunido |  |  |  |  |  |
| 1. Para hacer una investigación que ayudará a otros |  |  |  |  |  |
| 1. Para ayudar a los abogados a probar su caso en demandas que alegan que los productos médicos no son seguros |  |  |  |  |  |
| 1. Para obtener más información sobre las enfermedades que solo tiene un pequeño número de personas (combinando datos de muchos estudios de investigación) |  |  |  |  |  |

1. En términos generales, ¿con qué frecuencia puede confiar en otras personas?
   1. Siempre
   2. La mayor parte del tiempo
   3. Aproximadamente la mitad del tiempo
   4. De vez en cuando
   5. Nunca
2. ¿Qué grado de confianza tienen …?

|  | De ningún modo | Un poco | Una cantidad moderada | Bastante | Mucho |
| --- | --- | --- | --- | --- | --- |
| 1. Científicos en universidades y otras organizaciones sin fines de lucro |  |  |  |  |  |
| 1. Personas que viven con lesiones de la médula espinal |  |  |  |  |  |
| 1. Empresas que desarrollan productos médicos, como medicamentos recetados |  |  |  |  |  |
| 1. Doctores cuidando pacientes |  |  |  |  |  |
| 1. Compañías de seguros de salud |  |  |  |  |  |
| 1. Agencias gubernamentales |  |  |  |  |  |

1. ¿Cuál de las siguientes opciones describe mejor cómo se sentiría si le pidieran permiso para compartir los datos de la investigación individual anónima con personas ajenas al estudio de investigación en el que participó (suponiendo que su identidad esté bien protegida)? (MARCAR UNO)
   1. Se me debe pedir formalmente permiso para compartir mis datos, aparte de mi decisión de participar en el estudio de investigación. Mi permiso cubriría ampliamente cualquier uso potencial de mis datos en el futuro por parte de otros.
   2. Se me debe pedir formalmente permiso para compartir mis datos, aparte de mi decisión de participar en el estudio de investigación, cada vez que otros acceden a mis datos.
   3. No necesito proporcionar ningún tipo permiso para que otros accedan a mis datos más allá de consentir participar en el estudio original.
   4. No quiero que mis datos sean compartidos con personas ajenas al estudio de investigación.
2. Suponga que un estudio de investigación ya ha finalizado y que los participantes no fueron informados de que sus datos podrían ser compartidos. El equipo de investigación solo prometió a sus participantes que sus datos serian protegidos contra las brechas de seguridad. No hay forma de ponerse en contacto con los participantes ahora. ¿Cuál de los siguientes describe mejor su punto de vista? (MARCAR UNO)
   1. Los datos no deben compartirse.
   2. Está bien compartir los datos siempre que sean anónimos y no haya información de identificación adjunta, como mi nombre o fecha de nacimiento.
3. ¿Cuál es la razón más importante para preguntar a los participantes antes de compartir sus datos de la investigación individual anónima? (MARCAR UNO)
   1. Siempre existe algún riesgo para los participantes, incluso con buenas protecciones de seguridad.
   2. Es parte de mostrar respeto por los participantes.
   3. Ninguno; No es necesario consultar a los participantes.
   4. Otro (por favor escriba):
4. En términos generales, si se compartieron sus datos de la investigación individual anónima, ¿cree que debería recibir un reembolso monetario? Este pago sería separado de lo que haya recibido por la participación original en el estudio de investigación (si se proporcionó alguno).
   1. Sí
   2. No

Sistemas de Intercambio de Datos

 Lea las siguientes descripciones de 3 sistemas posibles para compartir datos de la investigación individual anónima. Luego se le preguntará qué sistema preferiría, si tuviera que elegir. Nuevamente, suponga que **los datos son anónimos**.

En **el Sistema de Patrocinador**, la compañía u otro patrocinador que pagó la investigación conserva los datos y considera las solicitudes para compartirlos. El patrocinador dice que compartirá los datos cada vez que alguien proponga un uso que pueda promover el conocimiento científico y acepte seguir los procedimientos de seguridad de datos.

En el **Sistema Independiente**, una organización independiente (como una universidad u otra organización sin fines de lucro) recibe los datos, establece un sitio web donde las personas pueden solicitarlos y considera las solicitudes. La organización dice que compartirá los datos cada vez que alguien proponga un uso que pueda promover el conocimiento científico y acepte seguir los procedimientos de seguridad de datos.

En el **Sistema de Acceso Abierto**, los datos se publican en un sitio web y cualquiera puede descargar los datos después de proporcionar su nombre y organización.

1. A fin de cuentas, ¿qué sistema para compartir datos de investigación preferiría?
   1. Sistema de Patrocinador
   2. Sistema Independiente
   3. Sistema de Acceso Abierto
   4. Ninguna de las anteriores
2. Al elegir ese sistema, ¿cuán importantes fueron los siguientes pasos para usted?

|  | No importante | Algo sin importancia | Ni importante ni sin importancia | Algo importante | Muy importante |
| --- | --- | --- | --- | --- | --- |
| a. Asegurarse de que se tomen decisiones justas sobre quién obtiene los datos |  |  |  |  |  |
| b. Asegurarse de que los datos se utilizan con fines legítimos |  |  |  |  |  |
| c. Tener un sistema confiable |  |  |  |  |  |
| d. Asegurarse de que el sistema proporciona una buena seguridad de datos |  |  |  |  |  |
| e. Asegurarse de que se siguen las reglas del sistema |  |  |  |  |  |
| f. Otro (por favor escriba): |  |  |  |  |  |

1. Para que se sienta cómodo compartiendo sus datos abiertamente. ¿Qué tan seguro (del 0 al 100%) tiene que estar usted de que su identidad no será revelada?
2. En general, ¿cómo cree que los potenciales beneficios de compartir datos de la investigación individual anónima sobrepasan los posibles negativos? (MARCAR UNO)
   1. Los negativos superan fuertemente los beneficios
   2. Los negativos superan moderadamente los beneficios
   3. Los negativos superan un poco los beneficios
   4. Beneficios y negativos son iguales
   5. Los beneficios superan un poco a los negativos
   6. Los beneficios superan moderadamente a los negativos
   7. Los beneficios superan fuertemente a los negativos
3. ¿La pandemia de COVID-19 ha modificado sus opiniones sobre el intercambio de datos?
   1. Estoy más dispuesto a compartir mis datos.
   2. No ha cambiado mis opiniones
   3. Estoy menos dispuesto a compartir mis datos.
   4. Inseguro
4. Si supiera que las características de su LME aumentaron su riesgo de re-identificación (por ejemplo, una forma rara de LME), ¿cambiaría eso su nivel de soporte para el intercambio de datos?
   1. Sí
   2. No
   3. No estoy seguro

Si hay algo que le gustaría explicar sobre su visión general, hágalo a continuación:

**ENCUESTA SOBRE LAS LESIONES DE MÉDULA ESPINAL**

Las siguientes preguntas se refieren a usted, su LME y su salud general, incluyendo qué tipo de LME tiene y cómo afecta su vida. Si se siente incómodo o no puede responder una pregunta, no dude en omitir la pregunta o detener la encuesta en cualquier momento.

1. ¿Dónde reside actualmente?
   1. Estados Unidos
   2. Canadá
   3. Otro (por favor escriba): ______________
2. ¿Con qué género te identificas?
   1. Mujer
   2. Hombre
   3. No binario / tercer género
   4. Prefiero auto describirme: ______________
   5. Prefiero no responder
3. ¿Cuál es el nivel más alto de educación formal que ha completado? (MARCAR UNO)
   1. Menos que la escuela secundaria
   2. Diploma de escuela secundaria
   3. Diploma o certificado de la escuela profesional, técnica o vocacional, o universidad o CEGEP
   4. Bachiller, Licenciatura o la Escuela de Magisterio
   5. Título de posgrado
   6. Prefiero no responder
4. ¿Con cuál de los siguientes grupos étnicos se identifica? (MARCA TODO LO QUE APLIQUE)
   1. Blanco
   2. Chino
   3. Primeras Naciones, Métis, Inuit, Indio Americano o Nativo de Alaska
   4. Asia meridional (por ejemplo, indio del este, paquistaní, esrilanquesa, etc.)
   5. Negro o afroamericano
   6. Latinoamericano
   7. Sudeste asiático (por ejemplo, vietnamita, camboyano, malasio, laosiano, etc.)
   8. Árabe
   9. Asia occidental (por ejemplo, iraní, afgano, etc.)
   10. Coreano
   11. Japonés
   12. Nativo de Hawái u otra isla del Pacífico
   13. Otro (por favor escriba): ______________
   14. No se
   15. Prefiero no responder
5. ¿Cuál es su edad actual (años)? ______________

**CLASIFICACIÓN DE LESIONES EN LA MÉDULA ESPINAL**

Esta sección le pregunta sobre su lesión de la médula espinal y las complicaciones derivadas de la lesión.

1. ¿Qué edad tenía cuando le ocurrió la lesión de la médula espinal (años)? ___________
2. ¿Cuántos años ha vivido con una lesión en la médula espinal?
   1. 1 año
   2. 2 años
   3. 3 años
   4. 4 años
   5. 5+ años
3. ¿Cuál de las siguientes opciones describe mejor dónde ocurrió su lesión de la médula espinal? (MARCAR UNO)
   1. Lesión en mi cuello (es decir, columna cervical)
   2. Lesión en la parte superior de la espalda (es decir, la columna torácica superior)
   3. Lesión en la parte media de la espalda (es decir, la columna torácica inferior)
   4. Lesión en la parte baja de la espalda (es decir, columna lumbar)
4. ¿Cuál de las siguientes opciones describe mejor la clasificación de su lesión medular? (MARCAR UNO)
   1. Cuadriplejia (también conocida como tetraplejia): una lesión que ha afectado sus brazos, manos, tronco y piernas
   2. Paraplejia: una lesión que ha afectado solo el tronco y las piernas (incluye la cauda equina)
5. ¿Cuál de las siguientes opciones describe mejor la causa de su lesión en la médula espinal? (MARCAR UNO)

a. Traumático: accidente de vehículo, caída, asalto, deportes, etc.

b. No traumático: tumor, infección, síndrome congénito, neurológico, columna degenerativa, accidente cerebrovascular dentro de la médula espinal (y no en el cerebro), complicación quirúrgica, etc.

1. Piensa en tu movilidad diaria. Para moverte, ¿generalmente camina o usa una silla de ruedas? ¿Cuál de las siguientes afirmaciones se adapta mejor a su forma principal de moverse? Si usa más de una manera, igualmente, marque ambas.

a. Camino sin la ayuda de un ayudante, herramienta o persona especial.

b. Camino con la ayuda de un asistente especial, herramienta o persona

C. Yo uso una silla de ruedas manual

d. Yo uso una silla de ruedas eléctrica o un scooter

e. Otro: ______________

1. A veces, una lesión de la médula espinal causa problemas secundarios a la lesión real. ¿Con qué frecuencia ha experimentado los siguientes problemas en las últimas 4 semanas?

|  | Nunca | De vez en cuando | Algunas veces | La mayor parte del tiempo | Todo el tiempo |
| --- | --- | --- | --- | --- | --- |
| 1. Disfunción sexual |  |  |  |  |  |
| 1. Dolor |  |  |  |  |  |
| 1. Infecciones del tracto urinario |  |  |  |  |  |
| 1. Contracturas articulares (una o más articulaciones que están congeladas y no se mueven) |  |  |  |  |  |
| 1. Problemas de hombro |  |  |  |  |  |
| 1. Incontinencia urinaria o intestinal |  |  |  |  |  |
| 1. Problemas de peso |  |  |  |  |  |
| 1. Problemas para dormir |  |  |  |  |  |
| 1. Problemas de codo o muñeca |  |  |  |  |  |
| 1. Úlceras por presión |  |  |  |  |  |
| 1. Fatiga |  |  |  |  |  |
| 1. Lesiones que se deben a una pérdida de sensibilidad en esa parte del cuerpo |  |  |  |  |  |
| 1. Aturdimiento o mareos. |  |  |  |  |  |
| 1. Estreñimiento |  |  |  |  |  |
| 1. Infecciones respiratorias |  |  |  |  |  |
| 1. Cálculos renales o vesicales |  |  |  |  |  |
| 1. Disreflexia autónoma (presión arterial alta repentina) |  |  |  |  |  |
| 1. Coágulos de sangre |  |  |  |  |  |

1. En general, ¿diría que su salud es: (MARCAR UNO)
   1. Excelente
   2. Muy buena
   3. Buena
   4. Justa
   5. Pobre
2. ¿Nos permitirá compartir sus datos anónimos de esta encuesta con otros investigadores? Si responde que no, NO compartiremos ninguna de sus respuestas. Si responde que sí, solo compartiremos sus datos **anónimos** y **no identificados** con otros investigadores, su dirección de correo electrónico NO se compartirá.
   1. Sí
   2. No

| Final |
| --- |

¡Gracias por tomarse el tiempo para completar esta encuesta! Si desea recibir una tarjeta de regalo de $10, responda al correo electrónico que le envió su enlace de encuesta único que indica que ha completado la encuesta y especifique si desea una tarjeta para:

Starbucks,

Tim Hortons,

Home Hardware,

Chapters / Indigo,

Winners

o Best Buy.

Si desea que la tarjeta de regalo se envíe a un correo electrónico diferente, especifíquelo en su mensaje.
