# Supplementary material for: Perspectives on Data Sharing in Persons With Spinal Cord Injury
Source: Neurotrauma Rep. 2023 Nov 9;4(1):781–9. doi: 10.1089/neur.2023.0035 (PMC10659015; doi:10.1089/neur.2023.0035)
Supplement: Supplemental data [file Suppl_TableS1.docx]

**Table S1: Concerns of data sharing**

|  | Not at all concerned (%) | Not very concerned (%) | Somewhat concerned (%) | Very concerned (%) | Did not respond (%) |
| --- | --- | --- | --- | --- | --- |
| Someone who is good with computers could identify the data | 67 (28.9) | 96 (41.4) | 48 (20.7) | 13 (5.6) | 8 (3.4) |
| People could be discriminated against if the information was linked back to them | 92 (39.7) | 60 (25.9) | 53 (22.8) | 18 (7.8) | 9 (3.9) |
| People could be embarrassed if the information was linked back to them | 86 (37.1) | 73 (31.5) | 44 (19.0) | 20 (8.6) | 9 (3.9) |
| People might use the data to do poor-quality science | 58 (25.0) | 76 (32.8) | 65 (28.0) | 23 (9.9) | 10 (4.3) |
| The information might be used in scientific projects that the participants wouldn’t approve of | 75 (32.3) | 80 (34.5) | 50 (21.6) | 16 (6.9) | 11 (4.7) |
| Some person or company could make a lot of money developing products using people’s information | 63 (27.2) | 89 (38.4) | 52 (22.4) | 18 (7.8) | 10 (4.3) |
| It could be harder to get people to agree to be in research studies if they know their data will be shared | 65 (28.0) | 81 (34.9) | 61 (26.4) | 14 (6.0) | 11 (4.7) |
| The information might be stolen | 49 (21.4) | 76 (33) | 69 (29.7) | 27 (11.6) | 11 (4.7) |
| Companies might use the information for marketing purposes instead of scientific purposes | 53 (23.8) | 70 (30.2) | 75 (32.3) | 26 (11.2) | 8 (3.4) |
| Scientists or companies could unfairly “free ride” on the work of others | 74 (31.9) | 85 (36.6) | 46 (19.8) | 16 (6.9) | 11 (4.7) |
| Scientists and companies might have less incentive to invest time and money in doing research studies | 63 (27.4) | 85 (36.6) | 57 (24.6) | 17 (7.3) | 10 (4.3) |
