# Supplementary material for: Perspectives on Data Sharing in Persons With Spinal Cord Injury
Source: Neurotrauma Rep. 2023 Nov 9;4(1):781–9. doi: 10.1089/neur.2023.0035 (PMC10659015; doi:10.1089/neur.2023.0035)
Supplement: Supplemental data [file Suppl_TableS2.docx]

**Table S2: Most concerning potential negative consequences of data sharing**

| Potential consequence | N (%) |
| --- | --- |
| People might use the data to do poor-quality science | 32 (13.8) |
| Companies might use the information for marketing purposes instead of scientific purposes | 29 (12.5) |
| The information might be stolen | 29 (12.5) |
| People could be discriminated against if the information was linked back to them | 23 (9.9) |
| The information might be used in scientific projects that the participants wouldn’t approve of | 20 (8.6) |
| People could be embarrassed if the information was linked back to them | 19 (8.2) |
| Some person or company could make a lot of money developing products using people’s information | 16 (6.9) |
| Scientists and companies might have less incentive to invest time and money in doing research studies | 16 (6.9) |
| It could be harder to get people to agree to be in research studies if they know their data will be shared | 13 (5.6) |
| Someone who is good with computers could identify the data | 11 (4.7) |
| Did not respond | 10 (4.3) |
| Other concerns | 8 (3.4) |
| Scientists or companies could unfairly “free ride” on the work of others | 6 (2.6) |
