# Supplementary material for: Perspectives on Data Sharing in Persons With Spinal Cord Injury
Source: Neurotrauma Rep. 2023 Nov 9;4(1):781–9. doi: 10.1089/neur.2023.0035 (PMC10659015; doi:10.1089/neur.2023.0035)
Supplement: Supplemental data [file Suppl_TableS3.docx]

**Table S3: How much would the following groups benefit from data sharing**

|  | Not at all (%) | A little (%) | A moderate amount (%) | A lot (%) | A great deal (%) | Did not respond (%) |
| --- | --- | --- | --- | --- | --- | --- |
| Scientists in universities and other not-for-profit organizations | 4 (1.7) | 8 (3.4) | 27 (11.6) | 69 (29.7) | 112 (48.3) | 12 (5.2) |
| People living with spinal cord injuries | 5 (2.2) | 16 (6.9) | 33 (14.2) | 63 (27.2) | 103 (44.4) | 12 (5.2) |
| Companies developing medical products, such as prescription drugs | 7 (3.0) | 8 (3.4) | 34 (14.7) | 83 (35.8) | 87 (37.5) | 13 (5.6) |
| Doctors taking care of patients | 5 (2.2) | 14 (6.0) | 22 (9.5) | 73 (31.5) | 105 (45.3) | 13 (5.6) |
| Health insurance companies | 8 (3.4) | 29 (12.5) | 62 (26.7) | 60 (25.9) | 61 (26.3) | 12 (5.2) |
| Government agencies | 6 (2.6) | 23 (9.9) | 64 (27.6) | 72 (31.0) | 53 (22.8) | 14 (6.0) |
