# Supplementary material for: Perspectives on Data Sharing in Persons With Spinal Cord Injury
Source: Neurotrauma Rep. 2023 Nov 9;4(1):781–9. doi: 10.1089/neur.2023.0035 (PMC10659015; doi:10.1089/neur.2023.0035)
Supplement: Supplemental data [file Suppl_TableS4.docx]

**Table S4: Anticipated benefits of data sharing**

|  | Not at all (%) | A little (%) | A moderate amount (%) | A lot (%) | A great deal (%) | Did not respond (%) |
| --- | --- | --- | --- | --- | --- | --- |
| Can help get answers to scientific questions faster using information that others have already gathered | 2 (0.9) | 8 (3.4) | 39 (16.8) | 80 (34.5) | 90 (38.8) | 13 (5.6) |
| Can help ensure that research dollars are spent as wisely as possible | 13 (5.6) | 14 (6.0) | 46 (19.8) | 87 (37.5) | 58 (25.0) | 14 (6.0) |
| Can lower the cost of developing new medical products | 9 (3.9) | 19 (8.2) | 48 (20.7) | 74 (31.9) | 65 (28.0) | 17 (7.3) |
| Can help people living with spinal cord injuries learn more about health problems that affect them | 4 (1.7) | 11 (4.7) | 32 (13.8) | 74 (31.9) | 97 (41.8) | 14 (6.0) |
| Can help scientists check the accuracy of research results announced by other scientists or companies (by redoing the analyses) | 5 (2.2) | 18 (7.8) | 35 (15.1) | 86 (37.1) | 74 (31.9) | 14 (6.0) |
| Can support learning about diseases that only a small number of people have (by combining data from many research studies) | 8 (3.4) | 16 (6.9) | 33 (14.2) | 74 (31.9) | 86 (37.1) | 15 (6.5) |
| Can discourage scientists and companies from hiding or distorting their research study results (by making it possible for others to check their analyses) | 12 (5.2) | 15 (6.5) | 50 (21.6) | 78 (33.6) | 63 (27.2) | 14 (6.0) |
| Can help lawyers prove their case in lawsuits claiming that medical products are unsafe | 28 (12.1) | 33 (14.2) | 71 (30.6) | 52 (22.4) | 33 (14.2) | 15 (6.5) |
| Can make sure people’s participation in research studies leads to the most scientific benefit possible | 3 (1.3) | 10 (4.3) | 33 (14.2) | 84 (36.2) | 88 (37.9) | 14 (6.0) |
