# Supplementary material for: Perspectives on Data Sharing in Persons With Spinal Cord Injury
Source: Neurotrauma Rep. 2023 Nov 9;4(1):781–9. doi: 10.1089/neur.2023.0035 (PMC10659015; doi:10.1089/neur.2023.0035)
Supplement: Supplemental data [file Suppl_TableS5.docx]

**Table S5: Most Important Potential Benefit of Data Sharing**

| Potential consequence | N (%*) |
| --- | --- |
| Total | 232 (100) |
| Can help get answers to scientific questions faster using information that others have already gathered | 58 (25.0) |
| Can help patients and groups of patients learn more about health problems that affect them | 41 (17.7) |
| Can make sure people’s participation in research studies leads to the most scientific benefit possible | 30 (12.9) |
| Can support learning about diseases that only a small number of people have (by combining data from many research studies) | 21 (9.1) |
| Can lower the cost of developing new medical products | 19 (8.2) |
| Can help scientists check the accuracy of research results announced by other scientists or companies (by re-doing the analyses) | 16 (6.9) |
| Can help ensure that research dollars are spent as wisely as possible | 16 (6.9) |
| Did not respond | 13 (5.6) |
| Can discourage scientists and companies from hiding or distorting their research study results (by making it possible for others to check their analyses) | 11 (4.7) |
| Other benefit | 4 (1.7) |
| Can help lawyers prove their case in lawsuits claiming that medical products are unsafe | 3 (1.3) |
