# Supplementary material for: Perspectives on Data Sharing in Persons With Spinal Cord Injury
Source: Neurotrauma Rep. 2023 Nov 9;4(1):781–9. doi: 10.1089/neur.2023.0035 (PMC10659015; doi:10.1089/neur.2023.0035)
Supplement: Supplemental data [file Suppl_TableS6.docx]

**Table S6: Overall perceptions of data sharing**

| Perception | N (%) |
| --- | --- |
| Benefits strongly outweigh the negatives | 103 (44.4) |
| Benefits moderately outweigh the negatives | 65 (28.0) |
| Benefits outweigh the negatives a little | 16 (6.9) |
| Benefits and negatives are equal | 19 (8.2) |
| Negatives outweigh the benefits a little | 9 (3.9) |
| Negatives moderately outweigh the benefits | 4 (1.7) |
| Negatives strongly outweigh the benefits | 3 (1.3) |
| Did not respond | 13 (5.6) |
