# Supplementary material for: Perspectives on Data Sharing in Persons With Spinal Cord Injury
Source: Neurotrauma Rep. 2023 Nov 9;4(1):781–9. doi: 10.1089/neur.2023.0035 (PMC10659015; doi:10.1089/neur.2023.0035)
Supplement: Supplemental data [file Suppl_TableS7.docx]

**Table S7: Trust in organizations and individuals involved in using health information**

| Characteristic | Not at all (%) | A little (%) | A moderate amount (%) | A lot (%) | A great deal (%) | Did not respond (%) |
| --- | --- | --- | --- | --- | --- | --- |
| Scientists in universities and other not-for-profit organizations | 0 (0.0) | 10 (4.3) | 57 (24.6) | 79 (34.1) | 74 (31.9) | 12 (5.2) |
| People living with spinal cord injuries | 4 (1.7) | 6 (2.6) | 41 (17.7) | 94 (40.5) | 75 (32.3) | 12 (5.2) |
| Companies developing medical products, such as prescription drugs | 8 (3.4) | 28 (12) | 106 (45.7) | 57 (24.6) | 21 (9.1) | 12 (5.2) |
| Doctors taking care of patients | 3 (1.3) | 10 (4.3) | 51 (22.0) | 100 (43.1) | 56 (24.1) | 12 (5.2) |
| Health insurance companies | 31 (13) | 63 (27) | 76 (32.8) | 33 (14.2) | 17 (7.3) | 12 (5.2) |
| Government Agencies | 12 (5.2) | 52 (22) | 93 (40.1) | 46 (19.8) | 15 (6.5) | 14 (6.0) |
