# Supplementary material for: Perspectives on Data Sharing in Persons With Spinal Cord Injury
Source: Neurotrauma Rep. 2023 Nov 9;4(1):781–9. doi: 10.1089/neur.2023.0035 (PMC10659015; doi:10.1089/neur.2023.0035)
Supplement: Supplemental data [file Suppl_TableS8.docx]

**Table S8: Data Sharing Permission**

| Characteristic | N (%) |
| --- | --- |
| I do not need to provide any permission for others to access my data beyond consenting to participate in the original study | 84 (36.2) |
| I should be formally asked for permission to share my data, separate from my decision to be involved in the research study. My permission would broadly cover any potential use of my data in the future by others | 78 (33.6) |
| I should be formally asked for permission to share my data, separate from my decision to be involved in the research study, every time my data is accessed by others | 52 (22.4) |
| I do not want my data shared with people outside of the research study | 6 (2.6) |
| Did not respond | 12 (4.2) |
