# Supplementary material for: Perspectives on Data Sharing in Persons With Spinal Cord Injury
Source: Neurotrauma Rep. 2023 Nov 9;4(1):781–9. doi: 10.1089/neur.2023.0035 (PMC10659015; doi:10.1089/neur.2023.0035)
Supplement: Supplemental data [file Suppl_TableS9.docx]

**Table S9: Reason for seeking permission**

| Characteristic | N (%) |
| --- | --- |
| It’s part of showing respect for participants | 102 (44.0) |
| There is always some risk to participants, even with good security protections in place | 70 (30.2) |
| Neither; it’s not necessary to consult participants | 33 (14.2) |
| Other | 14 (6.0) |
| Did not respond | 13 (5.6) |
