# Supplementary material for: Perspectives on Data Sharing in Persons With Spinal Cord Injury
Source: Neurotrauma Rep. 2023 Nov 9;4(1):781–9. doi: 10.1089/neur.2023.0035 (PMC10659015; doi:10.1089/neur.2023.0035)
Supplement: Supplemental data [file Suppl_TableS10.docx]

**Table S10: Data sharing when permission cannot be obtained**

| Characteristic | N (%) |
| --- | --- |
| It's OK to share the data as long as it is anonymous and there is no identifying information attached, such as my name or birthdate | 160 (69.0) |
| The data shouldn't be shared | 57 (24.6) |
| Did not respond | 15 (6.5) |
