# Supplementary material for: Perspectives on Data Sharing in Persons With Spinal Cord Injury
Source: Neurotrauma Rep. 2023 Nov 9;4(1):781–9. doi: 10.1089/neur.2023.0035 (PMC10659015; doi:10.1089/neur.2023.0035)
Supplement: Supplemental data [file Suppl_TableS11.docx]

**Table S11: Monetary reimbursement for data sharing**

| Characteristic | N (%) |
| --- | --- |
| No | 133 (57.3) |
| Yes | 87 (37.5) |
| Did not respond | 12 (5.2) |
