# Supplementary material for: Perspectives on Data Sharing in Persons With Spinal Cord Injury
Source: Neurotrauma Rep. 2023 Nov 9;4(1):781–9. doi: 10.1089/neur.2023.0035 (PMC10659015; doi:10.1089/neur.2023.0035)
Supplement: Supplemental data [file Suppl_TableS12.docx]

**Table S12: Level of comfort sharing personal data**

|  | Very uncomfortable (%) | Somewhat uncomfortable (%) | Neutral (%) | Somewhat comfortable (%) | Very comfortable (%) | Not applicable (%) | Did not respond (%) |
| --- | --- | --- | --- | --- | --- | --- | --- |
| Spinal cord injury details | 13 (5.6) | 5 (2.2) | 20 (8.6) | 17 (7.3) | 161 (69.4) | 3 (1.3) | 13 (5.6) |
| Locomotion ability | 11 (4.7) | 10 (4.3) | 9 (3.9) | 26 (11.2) | 154 (66.4) | 7 (3.0) | 15 (6.5) |
| Emotional health | 16 (6.9) | 10 (4.3) | 17 (7.3) | 45 (19.4) | 129 (55.6) | 2 (0.9) | 13 (5.6) |
| Sexual dysfunction | 21 (9.1) | 16 (6.9) | 22 (9.5) | 53 (22.8) | 96 (41.4) | 10 (4.3) | 14 (6.0) |
| Spasticity | 16 (6.9) | 12 (5.2) | 9 (3.9) | 21 (9.1) | 149 (64.2) | 12 (5.2) | 13 (5.6) |
| Pain | 12 (5.2) | 6 (2.6) | 15 (6.5) | 29 (12.5) | 151 (65.1) | 4 (1.7) | 15 (6.5) |
| Bladder/bowel concerns | 13 (5.6) | 19 (8.2) | 16 (6.9) | 32 (13.8) | 135 (58.2) | 4 (1.7) | 13 (5.6) |
| Joint contractures | 13 (5.6) | 9 (3.9) | 15 (6.5) | 23 (9.9) | 137 (59.1) | 21 (9.1) | 14 (6.0) |
| Shoulder, elbow, or wrist problems | 13 (5.6) | 6 (2.6) | 13 (5.6) | 22 (9.5) | 154 (66.4) | 11 (4.7) | 13 (5.6) |
| Metabolic disease | 11 (4.7) | 9 (3.9) | 23 (9.9) | 26 (11.2) | 112 (48.3) | 38 (16.4) | 13 (5.6) |
| Weight problems | 13 (5.6) | 9 (3.9) | 15 (6.5) | 34 (14.7) | 139 (59.9) | 8 (3.4) | 14 (6.0) |
| Heart or blood problems | 17 (7.3) | 5 (2.2) | 15 (6.4) | 27 (11.6) | 134 (57.8) | 21 (9.1) | 13 (5.6) |
| Trouble sleeping | 10 (4.3) | 4 (1.7) | 18 (7.8) | 29 (12.5) | 147 (63.4) | 10 (4.3) | 14 (6.0) |
| Respiratory problems | 11 (4.7) | 21 (9.1) | 12 (5.2) | 22 (9.5) | 130 (56.0) | 22 (9.5) | 14 (6.0) |
| Pressure ulcers | 13 (5.6) | 5 (2.2) | 11 (4.7) | 32 (13.8) | 138 (59.5) | 18 (7.8) | 15 (6.5) |
| Problems with brain function | 12 (5.2) | 12 (5.2) | 18 (7.8) | 20 (8.6) | 126 (54.3) | 31 (13.4) | 13 (5.6) |
| Fatigue | 11 (4.7) | 9 (3.9) | 18 (7.8) | 23 (9.9) | 153 (65.9) | 5 (2.2) | 13 (5.6) |
| Injuries due to loss of sensation | 14 (6.0) | 13 (5.6) | 11 (4.7) | 22 (9.5) | 149 (64.2) | 10 (4.3) | 13 (5.6) |
| Lightheadedness or dizziness | 12 (5.2) | 10 (4.3) | 18 (7.8) | 19 (8.2) | 136 (58.6) | 24 (10.3) | 13 (5.6) |

Regression results

For each outcome variable, an initial “full” model was tested, incorporating all the explanatory variables that could be associated with the outcomes. The list of explanatory variables consisted of the following:

| **Low trust in others**  **Fair or poor self-reported health status**  **College and/or graduate degree**  **Gender (Female)**  **Black or African American**  **Other non-white race**  **Over the age of 49**  **Country**  **Prior research experience** | **Very or somewhat concerned about one or more risks in the re-identification domain**  **Very or somewhat concerned about one or more risks in the information theft domain**  **Very or somewhat concerned about one or more risks in the misappropriation domain**  **Very or somewhat concerned about one or more risks in the threats to science domain** |
| --- | --- |

We then ran a second model that excluded the last 5 variables on the explanatory variable list, pertaining to the perceived trust and risk domains. The reason behind the exclusion was that these variables might be explainable by demographic characteristics.

If a variable achieved a p-value of 0.2 or less in either model 1 or 2, we included it in the final model for that outcome variable. This process was repeated for each of the outcome variable. Due to a lack of variables valid to run a full model, we were unable to run a final model for predictors of being somewhat or very unlikely to allow one’s own research data to be shared with doctors taking care of patients, or with scientists in universities and other non-for-profit organizations.

Regression results are presented below for models that predicted the following outcomes:

Table S13: Perceiving that the negative consequences of data sharing outweigh the potential benefits (response categories, from 7-point scale: “negative strongly outweigh the benefits”, “negatives moderately outweigh the benefits”, or “negatives outweigh the benefits a little”)
