# Supplementary material for: Perspectives on Data Sharing in Persons With Spinal Cord Injury
Source: Neurotrauma Rep. 2023 Nov 9;4(1):781–9. doi: 10.1089/neur.2023.0035 (PMC10659015; doi:10.1089/neur.2023.0035)
Supplement: Supplemental data [file Suppl_TableS13.docx]

**Table S13: Logistic Regression Results: Predictors of perceiving that the negative consequences of data sharing outweigh the potential benefits**

|  | OR | 95% CI |
| --- | --- | --- |
| Low trust in others | O.28 | 0.01-1.95 |
| College and/or graduate degree | 0.14** | 0.03-0.50 |
| Over 49 years of age | 0.25 | 0.03-1.14 |
| Participated in prior research | 0.09** | 0.01-0.43 |
| Very or somewhat concerned about reidentification | 0.29 | 0.07-1.14 |
| Very or somewhat concerned about misappropriation | 10.3* | 1.64-206 |
| Constant | 0.38 | 0.02-2.95 |

**=p*<0.05, **=*p*<0.01
